# CommWalker: correctly evaluating modules in molecular networks in light of annotation bias

**DOI:** 10.1093/bioinformatics/btx706

**Published:** 2017-11-03

**Authors:** M D Luecken, M J T Page, A J Crosby, S Mason, G Reinert, C M Deane

**Affiliations:** 1 Department of Statistics, University of Oxford, Oxford, UK; 2 Doctoral Training Centre, University of Oxford, Oxford, UK; 3 Department of Informatics, UCB Pharma, Slough, UK; 4 Immunology Therapeutic Area, UCB Pharma, Slough, UK

## Abstract

**Motivation:**

Detecting novel functional modules in molecular networks is an important step in biological research. In the absence of gold standard functional modules, functional annotations are often used to verify whether detected modules/communities have biological meaning. However, as we show, the uneven distribution of functional annotations means that such evaluation methods favor communities of well-studied proteins.

**Results:**

We propose a novel framework for the evaluation of communities as functional modules. Our proposed framework, CommWalker, takes communities as inputs and evaluates them in their local network environment by performing short random walks. We test CommWalker’s ability to overcome annotation bias using input communities from four community detection methods on two protein interaction networks. We find that modules accepted by CommWalker are similarly co-expressed as those accepted by current methods. Crucially, CommWalker performs well not only in well-annotated regions, but also in regions otherwise obscured by poor annotation. CommWalker community prioritization both faithfully captures well-validated communities and identifies functional modules that may correspond to more novel biology.

**Availability and implementation:**

The CommWalker algorithm is freely available at opig.stats.ox.ac.uk/resources or as a docker image on the Docker Hub at hub.docker.com/r/lueckenmd/commwalker/.

**Supplementary information:**

Supplementary data are available at *Bioinformatics* online.

## 1 Introduction

A functional module is defined as a group of interacting proteins that together perform one or more functions. They are thought to represent an important level of organization in biology (Hartwell *et al.*, 1999). The broad definition of a module has led to a variety of approaches for module detection. Generally, modules are found by performing community detection on protein interaction networks (PINs) (Lewis *et al.*, 2010; Luo *et al.*, 2007; Mete *et al.*, 2008; Pereira-Leal *et al.*, 2004; Spirin and Mirny, 2003), or other networks of integrated biological data (Cantini *et al.*, 2015; Chen and Yuan, 2006; Ji *et al.*, 2014; Mitra *et al.*, 2013). Due to high error rates in molecular networks (Hart *et al.*, 2006), inconsistencies between the many available community detection methods (Hric *et al.*, 2014; Tripathi *et al.*, 2016), and noise in orthogonal datasets such as gene expression (Bammler *et al.*, 2005; Irizarry *et al.*, 2005) an evaluation step is often added to the pipeline to determine which of the communities should be accepted as modules. It is this evaluation step that we address in this paper.

Detecting substructures in networks is an idea that predates functional modules, and thus a large collection of methods exist for detecting modules (Fortunato, 2010; Porter *et al.*, 2009). Most of these can be classified as community detection methods. Community detection methods aim to find groups of nodes that interact more with each other than with the rest of the network. Here, we use four different community detection approaches: two methods that detect overlapping communities [Link Clustering (Ahn *et al.*, 2010) and BigCLAM (Yang and Leskovec, 2013)], and two methods that detect exact partitions [configuration model and constant potts model Modularity Maximization (Blondel *et al.*, 2008; Reichardt and Bornholdt, 2006; Traag *et al.*, 2011)]. The first three of these methods have been previously applied to PINs (Ahn *et al.*, 2010; Lewis *et al.*, 2010; Yang and Leskovec, 2013).

To evolve the concept of a community into a biologically meaningful functional module, module evaluation methods can be used. These methods use functional annotations, such as those sourced from the Gene Ontology (GO) (Ashburner *et al.*, 2000), to compute the functional homogeneity of proteins grouped into communities. The two main approaches to calculate functional homogeneity are functional enrichment and semantic similarity. Functional enrichment calculates the significance of an annotation in a community based on its prevalence compared to a random community of the same size (Ahn *et al.*, 2010; Huang *et al.*, 2009; Mete *et al.*, 2008). In contrast, semantic similarity measures use the relationships between annotations associated with proteins provided by the GO to compute a similarity score (Guzzi *et al.*, 2012; Pesquita *et al.*, 2009).

While these methods are widely used, it is often overlooked that the distribution of functional annotations across networks is heterogeneous. In the same way that research focus affects the topological structure of PINs (von Mering *et al.*, 2002; Rual *et al.*, 2005; Schaefer *et al.*, 2015), it also affects which proteins amass functional annotations (Pesquita *et al.*, 2008). This phenomenon and its effects are well-described in the field of gene function prediction (Greene and Troyanskaya, 2012; Myers *et al.*, 2006; Pavlidis and Gillis, 2012; Schnoes *et al.*, 2013). Yet, how annotation bias manifests itself in module detection remains to be investigated. Previous studies have shown that the number of functional annotations affect semantic similarity measures (Pesquita *et al.*, 2008; Wang *et al.*, 2010). Following on from these results, we demonstrate the consequences for module evaluation. Based on an analysis of PINs, we show that annotation heterogeneity leads to a preference of module evaluation for communities of well-studied proteins using both semantic similarity and functional enrichment. We propose the CommWalker module evaluation framework to counteract this bias.

CommWalker uses short random walks to sample the local network environment of a community and adjusts the stringency of the evaluation accordingly. In this way, CommWalker achieves a greater sensitivity in poorly studied network regions, while maintaining stringent module evaluation for well-studied communities. While random walks have been frequently used in community detection (Fortunato, 2010; Jeub *et al.*, 2015) and network analysis (Boccaletti *et al.*, 2006), their use in module evaluation is to our knowledge novel. To demonstrate CommWalker’s efficacy, we compare its perfomance with the semantic similarity measures simUI (Gentleman, 2005), simGIC (Pesquita *et al.*, 2008), and the Pandey method (Pandey *et al.*, 2008), all of which have previously been used for PIN analysis (Pesquita *et al.*, 2008; Lewis *et al.*, 2010).

## 2 Materials and methods

### 2.1 Protein interaction networks

In order to assess the performance of CommWalker on different types of protein interaction datasets, we downloaded two characteristically different human networks: HINT-P and BioGrid-AP (cf. Table 1).

Human protein interaction data were downloaded from the HINT (Das and Yu, 2012) (retrieved Aug. 2015) and BioGrid (Stark *et al.*, 2006) (retrieved Aug. 2015) databases. The data were divided into two categories: association data (A), and physical association data (P), which are broadly defined by PSI-MI classifiers MI: 0914 for A-type data, and MI: 0407 and MI: 0915 for P-type data (Côté *et al.*, 2010). While the HINT database assigns co-complex (A-type) or binary (P-type) labels to interactions, the BioGrid dataset was split into A-type and P-type interactions by experimental evidence codes after (Lewis *et al.*, 2010). Further filters were applied to the datasets to include only interactions between human proteins, exclude self-interactions and exclude any nodes not connected to the largest connected component of the PINs. Network statistics of the two datasets are shown in Table 1.

**Table 1.** Network statistics for HINT-P and BioGrid-AP

Network	Nodes	Edges	Density	Avg. Degree
HINT-P	10 927	49 301	0.00083	9.02
BioGrid-AP	15 405	165 343	0.00139	21.47

*Note*: BioGrid-AP is the larger network with a higher density. The PINs overlap in 10 617/10 927 possible nodes and 40 853/49 301 possible edges.

HINT-P uses only P-type interactions that have been reported by at least two independent sources (Das and Yu, 2012) and is a comparatively small but high confidence dataset. BioGrid-AP uses A-type and P-type interaction data and is thus more comprehensive, but likely to have a high false positive rate.

### 2.2 Community detection

The communities evaluated by CommWalker were generated by four different community detection methods: two non-overlapping methods [configuration model Modularity Maximization (Blondel *et al.*, 2008; Reichardt and Bornholdt, 2006) and Constant Potts model Modularity Maximization (Traag *et al.*, 2011; Blondel *et al.*, 2008)] and two overlapping community detection methods [Link clustering (Ahn *et al.*, 2010) and BigCLAM (Yang and Leskovec, 2013)]. These methods represent different approaches to the community detection problem (Fortunato, 2010) and are elaborated on in Appendix A in the Supplementary Material.

As functional modules can be found at different scales of organization (Lewis *et al.*, 2010), we intentionally avoid partitioning our networks in only a single way. Instead, we selected community detection methods that either have an inbuilt resolution parameter or a parameter which can be used as a proxy for the resolution of a network partition. Using multi-resolution community detection we generated a multitude of network partitions according to each method, thus covering a wide spectrum of possible communities from the PINs used. The inbuilt resolution parameter, *S*, was used for Link clustering (Ahn *et al.*, 2010) (https://github.com/bagrow/linkcomm, retrieved June 2014) at 121 values evenly spanning the interval S∈[0,0.6]. For Modularity Maximization community detection, we used the adaptation of Modularity from (Reichardt and Bornholdt, 2006) as implemented in the Louvain algorithm (Blondel *et al.*, 2008) (https://launchpad.net/louvain, retrieved April 2014) which includes the resolution parameter, *γ*. Network partitions were generated at 51 resolutions spanning the interval $\gamma \in [10^{-1},10^{3}]$ for the configuration model and $\gamma \in [10^{-4},10^{0}]$ for the Constant Potts model, with resolutions evenly spaced on a logarithmic scale. As BigCLAM (Yang and Leskovec, 2013) (http://snap.stanford.edu, retrieved June 2014) does not have an inbuilt resolution parameter, we used the number of communities to be fitted, *K*, as a proxy for the resolution. Network partitions were generated at 101 *K* values evenly spanning the interval K∈[1,5001]. Using this proxy, network partitions at neighbouring resolutions only differ in the added 50 communities that are fitted at the higher resolution. Thus the number of proteins in functionally significant communities increases with the parameter *K*, so that a maximum of the number of proteins in functionally significant communities is trivially found at the highest *K*. The non-backtracking line search was parametrized at α=β=0.9 for the BigCLAM algorithm to optimize for partition quality over speed.

For practical purposes, we limit the size range of communities of interest to 6–35. Using this limitation, the communities proposed as modules are viable to be experimentally tested, and are unlikely to be trivial associations.

### 2.3 Semantic similarity

To quantify the similarity between proteins three semantic similarity measures based on Gene Ontology (GO) Biological Process (BP) annotations [human assocation data from http://www.geneontology.org (Ashburner *et al.*, 2000), retrieved July 2015; GO ontology retrieved August 2015] were used. All GO term associations with evidence codes ‘IPI’ and ‘RCA’ were filtered out, as these associations are inferred based on protein interactions or omics data that may include protein interaction networks themselves. This filtering ensures that there is no data circularity. Further filtering was applied to ‘ND’ evidence codes, denoting no evidence for specific BP association, and ‘NOT’-qualifiers, which denote negative associations.

Following reviews of semantic similarity measures in protein interaction networks and other biological applications (Guzzi *et al.*, 2012; Mazandu and Mulder, 2014; Pesquita *et al.*, 2009), we chose a semantic similarity measure developed by Pandey *et al.* (Pandey *et al.*, 2008), and two popular and well-reviewed measures: simUI (Gentleman, 2005) and simGIC (Pesquita *et al.*, 2008). These three similarity measures all compute the functional similarity of proteins, *ρ*, based on the intersection of GO term sets each protein is associated with (cf. details in Appendix A in the Supplementary Material). For consistency, the figures presented in the main paper use the Pandey method, while results for simUI and simGIC can be found in the Supplementary Material.

Using these semantic similarity measures, the biological relevance of a community can be quantified via its functional homogeneity. The functional homogeneity is calculated by taking the average of the *ρ* scores of all protein pairs in a community. Proteins without functional annotations are ignored in this calculation.

The functional homogeneity of a community can be used to assess its significance by comparing it to a background semantic similarity value. Commonly used background values are related to the mean or median of the functional similarities of interacting proteins in a PIN (Lewis *et al.*, 2010).

### 2.4 Gene co-expression analysis

Gene co-expression analysis was performed using Genotype Tissue Expression (GTEx) project data (Version 6, RPKM format, from www.gtexportal.org/home/datasets, retrieved November 2015) (Lonsdale *et al.*, 2013). These data comprise of over 8500 tissue-specific, whole genome RNA-Seq samples which were extracted postmortem from human donors and prepared according to the same protocol. The data were retrieved in a processed format, in which expression values are reported per kilobase of transcript per million reads. Transcript IDs (Ensembl Gene IDs) were mapped to the PIN gene IDs (Entrez IDs) using the Ensembl release 82 Biomart tool (Cunningham *et al.*, 2015) and the expression profiles that were mapped to the same gene ID were averaged.

The level of co-expression of two genes was evaluated by taking the absolute value of the Pearson correlation coefficient of the genes’ expression profiles following (Langfelder and Horvath, 2008). The co-expression scores were then combined into a community co-expression score by taking the average of all pairwise co-expression scores in the community after (Jansen *et al.*, 2002). The distribution of these co-expression scores was used to compare communities evaluated as functionally significant by different methods.

To quantify how easily we can differentiate between community and random background co-expression, we computed the overlap between the co-expression score distribution of communities and a random background co-expression score distribution. Random background co-expression was generated by performing 1000 short random walks of length six from each node in HINT-P and BioGrid-AP and computing their co-expression scores. The overlap score between the two distributions was quantified by setting a threshold based on the community score distribution and calculating the proportion of random walks whose co-expression score exceeds this threshold. This threshold was set to the 25% quantile of the community co-expression score distribution (cf. Appendix E in the Supplementary Material, where we also discuss alternative thresholding).

## 3 Results

### 3.1 Inspection bias

PINs are noisy and incomplete, and the extent to which a given protein has been studied affects its representation in the network. For example, we know that well-studied proteins tend to have a higher degree in PINs (Rual *et al.*, 2005; Schaefer *et al.*, 2015; von Mering *et al.*, 2002). Similarly, better studied proteins tend to have more functional annotations. Previous work has shown that the number of functional annotations affects quantifications of functional similarity based on semantic similarity (Wang *et al.*, 2010). Here, we show how this effect also impacts functional module evaluation.

The impact of this heterogeneity of annotation on module evaluation can be analysed by testing for correlation between the functional homogeneity of ‘modules’ and how well-studied their components are. To perform this investigation independently of module detection methods, we used short random walks from each protein (node) in a PIN to represent random proxy modules. We quantified the research focus by using as a score the fraction of nodes that are functionally annotated (beyond the root biological process annotation) in a proxy module, and the functional homogeneity of this proxy module via four common module evalution methods: three semantic similarity measures (simUI, simGIC, and the Pandey method), and functional enrichment (Huang *et al.*, 2009) (cf. Methods). To obtain scores for each protein, these module-based measurements were mapped back to individual nodes by averaging the research focus scores as well as the functional homogeneity scores which are calculated on random walks started at the same node.

**Fig. 1.**
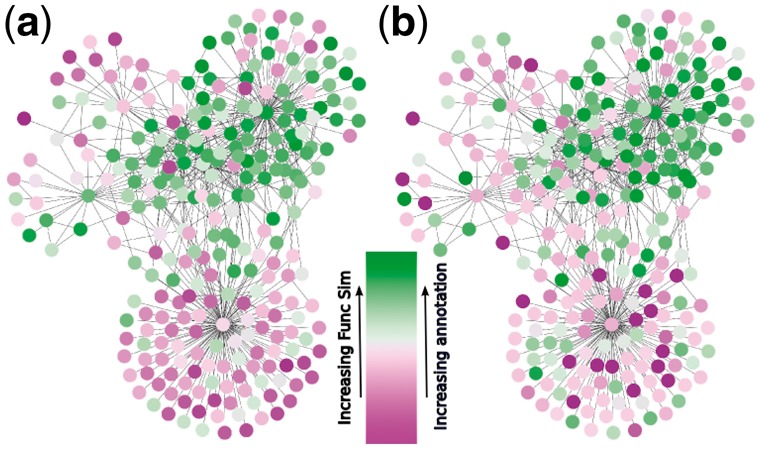
Semantic similarity and research focus correlation. The correlation between semantic similarity and research focus is shown on a subgraph of HINT-P, generated by taking all nodes connected to the gene FAT1 through at most two intermediary genes. (**a**) is coloured by the average Pandey method functional homogeneity in size 3 proxy modules around the proteins, and (**b**) is coloured by the research focus score. Regions of high functional similarity with the environment (‘Func Sim’) correlate with regions with strong research focus

Performing 10 000 random walks from each node, we found that research focus and functional homogeneity is correlated across the two PINs and four functional similarity measures investigated (cf. Appendix B in the Supplementary Material for further details). An example of this correlation for a subnetwork of HINT-P using the Pandey method (cf. Methods) is shown in Figure 1. This figure also shows that research focus appears to create regions of high and low functional homogeneity in PINs.

Our analysis shows that nodes may be evaluated as functionally similar to random nodes in their neighbourhood in regions of high functional homogeneity. Thus, communities in these regions will be more likely to be evaluated as highly functionally homogeneous, and vice versa in poorly studied regions, which biases module evaluation results. To counteract this bias, it is necessary to take the network region into account when evaluating modules. For this purpose, we have developed CommWalker, which uses short random walks to sample a community’s local network environment to put its functional homogeneity into the correct context. CommWalker is designed to counteract the overestimation of the functional homogeneity of communities in well-studied environments, while allowing for positive evaluation of modules in poorly studied network regions.

### 3.2 CommWalker

CommWalker is a method framework and can be used with any semantic similarity measure defined between nodes. CommWalker uses these measures to calculate a community significance score, which is obtained by relating the functional homogeneity of the community to the functional homogeneity distribution of the community’s local network background.

**Fig. 2.**
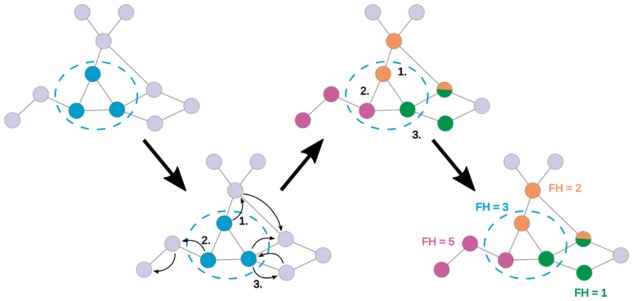
Schematic diagram of the methodology behind CommWalker. The local network area is sampled by random walks from the community nodes (dark blue nodes). Random walks are terminated when they have visited *N_C_* nodes, where *N_C_* is the community size (here *N_C_* = 3). The terminated random walks represent proxy communities (orange, magenta, and green) whose functional homogeneity values give the local background distribution in which to interpret the community functional homogeneity. At a functional homogeneity score of 3, the *T*-value of the blue community is $\frac{1+1}{3+1}=0.5$, as one proxy community has a higher functional homogeneity. While functional homogeneity is generally calculated by averaging GO annotation based functional similarity scores between all protein pairs in a community (see Methods), the values shown here are chosen for illustration only

Figure 2 is a schematic diagram of the CommWalker evaluation methodology. CommWalker performs short random walks from each node in a community. A random walk is terminated when it has visited *N_C_* distinct nodes, where *N_C_* is the number of nodes in the community. Each random walk can therefore be interpreted as an alternative choice of community in this local network environment. Using the functional homogeneity values of the random walks, the tail-value, or *T*-value, is computed to represent the significance of the community in its environment. The *T*-value is calculated by $T=\frac{m+1}{M+1}$, where *m* denotes the number of random walks with a functional homogeneity higher than that of the initial community, and *M* is the total number of random walks from this community. The *T*-value is thus the fraction of the background distribution in the upper tail as defined by the community functional homogeneity value (cf. Appendix C in the Supplementary Material).

In the CommWalker algorithm the number of random walks started per node is calculated based on the size of the community to ensure each community is sampled to a similar extent. We investigated how many random walks were needed to generate a stable background distribution, and thus a stable *T*-value, for a community. The best trade-off between algorithm run-time and *T*-value stability was found at a *T*-value standard error of ≈ 0.005. Further details on this analysis and the CommWalker implementation can be found in Appendix C in the Supplementary Material.

### 3.3 CommWalker module analysis

CommWalker is designed as a module evaluation framework which counteracts annotation bias to allow for fair module evaluation even in poorly-studied network regions. As such, the efficacy of this framework can be investigated via the communities CommWalker accepts as modules, specifically via their distribution in PINs. In order to compare module evaluation using CommWalker to module evaluation without it, we chose qualitatively similar module acceptance thresholds for *T*-value and functional homogeneity scores. At a *T-*value of 0.5 approximately half of the random walks have a functional homogeneity at least as high as that of the community. Similarly, the median of the semantic similarities of interacting proteins is the smallest value where half of the interacting proteins in the network have a semantic similarity at least this high. These thresholds were chosen to ensure the best comparison between evaluation approaches rather than for the purpose of rigorous biological validation.

We performed module evaluation on communities from four multi-resolution community detection algorithms and two PINs using the three aforementioned semantic similarity measures for functional homogeneity evaluation (cf. Methods). Community *T*-values and functional homogeneities were computed using each of the semantic similarity measures. Communities whose functional homogeneity exceeded our threshold were regarded as accepted by functional homogeneity evaluation without CommWalker, and likewise those communities with a *T*-value below our threshold were labeled CommWalker accepted. In this way, communities were divided into four sets: accepted by both methods, accepted only by CommWalker, accepted only by functional homogeneity, and rejected by both methods. Communities in these sets were analysed for their size, their level of annotation, and the level of annotation of their local environment. Across networks, community detection methods, and semantic similarity measures, we found that modules accepted only by functional homogeneity without CommWalker tend to be small, well-studied, and in well-studied environments in contrast to those modules accepted only by CommWalker which instead are more broadly distributed in these statistics (cf. Appendix D in the Supplementary Material).

**Fig. 3.**
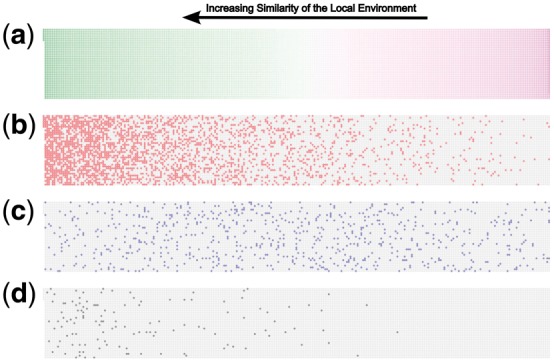
Comparison of the environments of proteins in communities identified as biologically relevant by CommWalker and functional homogeneity. Nodes in HINT-P were ordered by their Pandey semantic similarity with nodes in their vicinity as shown in (**a**). Communities were generated by configuration model Modularity Maximization at the resolution where the maximum number of proteins are found in functionally significant communities by a *T*-value threshold of 0.5 (log resolution = 1.80, cf. Appendix D in the Supplementary Material). On this network layout the proteins in communities identified as functionally significant by both CommWalker and functional homogeneity are shown in red (**b**), by only CommWalker and not functional homogeneity in blue (**c**), and by only functional homogeneity and not CommWalker in black (**d**). The further left the coloured nodes are, the higher the semantic similarity of their environment

Figure 3 shows CommWalker’s higher sensitivity in low functional homogeneity regions, which correspond to less well-studied regions of the network. For ease of visualization, non-overlapping community data were used from configuration model Modularity Maximization on HINT-P data, in conjunction with Pandey semantic similarity. In Figure 3, the proteins are ordered by their semantic similarity with their local environment, measured as described in the *Inspection bias* Section. Proteins towards the left have higher similarity with their environment and will thus tend to be better studied. On this layout, we show the distribution of proteins in communities that were accepted as modules by both methods (Fig. 3b), only by CommWalker (Fig. 3c), and only by functional homogeneity (Fig. 3d). Proteins in modules accepted by the standard functional homogeneity approach (Fig. 3b, d) tend to be distributed towards the well-studied left side of the figure. In contrast, modules accepted only by CommWalker (Fig. 3c) have a broader distribution, reaching into the poorly studied protein regions. In the data presented in Figure 3, CommWalker accepted modules contain 30.2% of the proteins in the PIN, and modules accepted by functional homogeneity contain 24.6% of the proteins. However, among proteins with functional similarity scores in the bottom quartile (right hand quarter of the panels in Fig. 3) functional homogeneity accepted modules include 3.6% of the proteins, while CommWalker accepted modules include 8.6%. All of the poorly studied functional homogeneity accepted modules are also accepted by CommWalker. This behaviour is also observed for poorly-studied proteins defined by research focus scores in the bottom quartile (functional homogeneity 10.6% and CommWalker 17.2% proteins in accepted modules, overlap of 10.2% of proteins).

Using non-overlapping community detection methods (configuration model and Constant Potts model Modularity Maximization) for both PINs and all three semantic similarity measures produced similar results (cf. Appendix D in the Supplementary Material). We further observed that the increased sensitivity in poorly-studied network regions evident from this analysis allows CommWalker to accept a greater number of communities as modules across most datasets investigated. We also verified that CommWalker prioritizes different communities to functional homogeneity irrespective of the threshold used (cf. Appendix D in the Supplementary Material). These results suggest that CommWalker allows for positive module evaluation even in network regions that were previously obscured by lack of annotation.

### 3.4 Computational module validation

CommWalker’s greater sensitivity in poorly-studied network regions results from an increased leniency in module evaluation in these regions. Under the assumption that functional module detection should span PINs as every protein performs a function, this leniency is theoretically warranted. Practically, it may however be the case that community detection fails in poorly-studied network regions, due to greater error rates in local network topology compared to well-studied regions. We thus performed systematic validation by comparing module co-expression between CommWalker and functional homogeneity accepted modules. Module co-expression measures how similar the expression profiles of genes in the same module are across human tissue samples (cf. Methods).

**Fig. 4.**
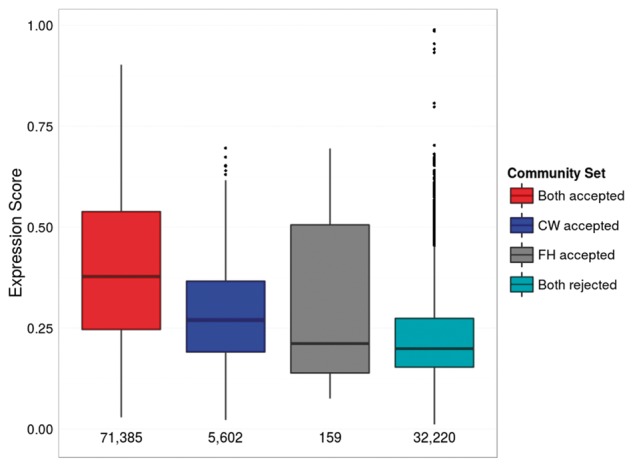
Comparison of community evaluation methods by gene co-expression. Link clustering was used to partition BioGrid-AP into communities at multiple resolutions. Communities across all resolutions were divided into sets based on whether they were evaluated as functionally significant, depicted from left to right: by both methods (‘Both accepted’; red), by only CommWalker (‘CW accepted’; blue), by only functional homogeneity (‘FH accepted’; grey), or by neither method (‘Both rejected’; turquoise) based on Pandey semantic similarity. These sets are compared in a boxplot of distributions of their community expression scores (cf. *Methods*) for communities of size 6 - 35. This size range allows us to exclude protein sets that represent module components such as small complexes as well as aggregations of modules that perform multiple functions. The number of data points in each community set is shown under the respective boxplot. The number of communities only accepted by CommWalker is ≈ 35 times as large as the number of communities only accepted by functional homogeneity. As shown by the median values of the distributions, communities only accepted by CommWalker have higher co-expression scores than communities only accepted by functional homogeneity. This result is further reflected in their overlap scores of 13.5%, 24.7%, 40.7%, and 35.6% in order of depiction (Color version of this figure is available at *Bioinformatics* online.)

As the relationship between gene co-expression and functional relatedness is not straightforward (Lee *et al.*, 2004; Li and Biggin, 2015; van Noort *et al.*, 2003; Zhou *et al.*, 2002), we do not expect that the four community detection methods necessarily capture gene co-expression. In order to perform the validation on a dataset that best captures co-expressed genes, we evaluated how much the community sets in each dataset differ from random walk co-expression. This evaluation is performed by computing the overlap between the co-expression score distributions of communities and random walks (cf. Methods). Overlap scores range from 0% indicating a strong co-expression signal in communities, to 100% indicating random co-expression. Link clustering on BioGrid-AP was found to best capture co-expression (cf. Appendix E in the Supplementary Material), with the only overlap score below 15% obtained for those communities accepted by both methods using the Pandey semantic similarity measure (Fig. 4).

Figure 4 shows that modules accepted by only CommWalker exhibit a distinctly higher level of co-expression than those accepted only by functional homogeneity. Indeed, modules only accepted by functional homogeneity have a similar median co-expression level than those rejected by both methods on this dataset. While Figure 4 does not provide conclusive evidence that all modules accepted by CommWalker are correct, it does suggest that CommWalker accepted modules are at least of a similar quality to modules accepted by commonly used functional homogeneity approaches.

We further performed a more detailed investigation of the largest module (in the Link clustering, BioGrid-AP, Pandey method dataset) only accepted by CommWalker at a stricter T-value threshold of 0.25, and a stronger functional homogeneity rejection threshold (FH < 5, instead of median semantic similarity of interacting proteins at 6.10552) in the above dataset. This module contains the TRAPP proteins TRAPPC2, TRAPPC3L, TRAPPC4, TRAPPC6B, TRAPPC8, TRAPPC10, and TRAPPC12, which are implicated in vesicle transport (Scrivens *et al.*, 2011). Despite its coherent functional description, it is only relatively poorly annotated at an average of 43.71 functional annotations per protein, compared to a mean of 89.85 functional annotions for proteins in BioGrid-AP with a standard deviation of 33.91. A similar investigation was performed for a module only accepted by functional homogeneity, whose proteins showed a more tenuous relationship (cf. Appendix E in the Supplementary Material). The prioritization of the TRAPP module despite its poor functional annotation exemplifies CommWalker’s ability to overcome poor functional annotation in module evaluation.

## 4 Discussion

Currently, a complete picture of protein function with corresponding functional annotation is not available. Thus, it is important to consider the effect of the distribution of annotations on the network in module evaluation. We have developed CommWalker, a module evaluation framework that takes this heterogeneity of annotation into account. CommWalker allows for an increased sensitivity in poorly-studied network regions, without sacrificing its ability to faithfully evaluate well-studied communities. Through this increased sensitivity CommWalker accepted modules have the potential to uncover functional structure in network regions where such advances are most needed. It is indeed in the network regions we know least about, that computational approaches have the greatest potential.

The most common method of evaluating communities or validating modules is functional enrichment (Ahn *et al.*, 2010; Huang *et al.*, 2009; Mete *et al.*, 2008). Functional enrichment evaluates functional significance based on how unlikely it is to obtain observed frequencies of GO terms given a random GO-term distribution over the network. However, we have shown that GO terms are not randomly distributed across the network. It is possible to use the CommWalker framework to overcome this limitation. Similar to the semantic similarity approach, the frequency of a GO-term can be related to the frequencies of this GO-term in random walks from the community to evaluate its significance.

In the same way that annotation bias affects module evaluation, biases in functional annotations also affect gene function prediction (Greene and Troyanskaya, 2012; Lee and Marcotte, 2009; Pavlidis and Gillis, 2012; Schnoes *et al.*, 2013). While the annotation bias that poses a problem for module evaluation is a skewed and clustered distribution of annotation specificity across PINs, the bias effect on gene function prediction most commonly described in the literature arises from a large coverage of unspecific annotations which are propagated through the network (functional bias, e.g. Schnoes *et al.*, 2013; Lee and Marcotte, 2009). Both biases arise from a heterogeneous distribution of annotation, yet their emphasis differs. One reason for this different focus is that gene function prediction is a local network process [e.g. guilt-by-association (Pavlidis and Gillis, 2012)] which is better equipped to deal with local differences in annotation across PINs, than module evaluation which compares modules to a global reference. Given that effects of highly annotated genes on function prediction have been reported (Pavlidis and Gillis, 2012), it is likely that the clustering of functional annotations into regions as described in this paper will also affect gene function prediction. Further work is necessary to understand the extent of this effect.

Here, we have demonstrated CommWalker’s performance using three popular semantic similarity measures on a variety of biological datasets. The CommWalker framework can however be used to improve module evalaution in conjuction with any semantic similarity measure, and is thus applicable to a wide range of node evaluation problems. While we have demonstrated its use in biological applications, CommWalker may likewise be applicable to social networks that exihibit a skewed distribution of annotations used for evaluation. For example, friendship groups in a network may be validated based on the results of a questionnaire. If the questionnaires were taken seriously to different degrees in each friendship group, CommWalker may be able to validate friendship groups even among individuals who only half-heartedly responded to questions.

As social network communities can be much larger than those in biological networks, it may be beneficial to rein in the random walks for these applications. The larger the community, the further into the network the random walks may sample – past what could be considered the local environment. Random walk methods such as ‘random walk with restart’ (Tong *et al.*, 2006) can be implemented to ensure local sampling.

Whether in social network applications using questionnaires, or in biological applications using functional annotations, it is always important that some annotation exists. While CommWalker can overcome lack of annotation to a certain extent, it is only capable of amplifying an existing signal. Thus, overall CommWalker is expected to accept more well-studied than poorly studied modules. The contribution of the CommWalker framework is that we can shift the balance further into poorly-studied regions of PINs. Continuous improvement of the coverage of functional annotations will introduce more functional signal into the data and thus further increase the benefits of CommWalker module evaluation. Nonetheless, already now CommWalker has the potential to uncover novel biology.

## Supplementary Material

Supplementary Data
