# Supplementary material for: CommWalker: correctly evaluating modules in molecular networks in light of annotation bias
Source: Bioinformatics. 2017 Nov 3;34(6):994–1000. doi: 10.1093/bioinformatics/btx706 (PMC5860269; doi:10.1093/bioinformatics/btx706)
Supplement: Supplementary Data [file btx706_supplementary_information_rver1.pdf]

Supplementary Information

MD Luecken, MJT Page, AJ Crosby, S Mason, G Reinert, and CM Deane

Contents

1 Appendix A: Methods **1**
1.1 Community Detection Methods 1
1.2 Semantic Similarity 3

2 Appendix B: Inspection Bias Results **5**

3 Appendix C: CommWalker Implementation Details **6**

4 Appendix D: Module Analysis Results **9**
4.1 Coverage of accepted modules 9
4.2 Module prioritization 9
4.3 Module Statistics 10

5 Appendix E: Module Validation Results **35**

1 Appendix A: Methods

1.1 Community Detection Methods

Community detection is a tool which partitions a network into groups of nodes which interact more with each other than with the rest of the network. These groups represent connected substructures, so-called communities, within the network based on its topology and edge weights.

Communities can be detected on different scales. In a molecular network a community may consist of ribosome related proteins, while such a community will contain groups of ribosomal subunit proteins, and groups of precursors to and processors of ribosomal subunits (Lewis *et al.*, 2010). A further example of this scale effect on HINT-P data can be seen in Fig. S.1.

Community detection methods can be subdivided into those that assign each protein to a single community (non-overlapping), and those that can assign proteins to multiple communities (overlapping). Here we use two non-overlapping (configuration model Modularity Maximization (Blondel *et al.*, 2008; Reichardt and Bornholdt, 2006) and Constant Potts model Modularity Maximization (Blondel *et al.*, 2008; Traag *et al.*, 2011)) and two overlapping (Link clustering (Ahn *et al.*, 2010) and BigCLAM (Yang and Leskovec, 2013)) community detection methods to partition PINs.

The non-overlapping community detection methods used here are based on the concept of Modularity. Modularity is a measure proposed by Newman and Girvan in 2004 (Newman and Girvan, 2004), which describes the strength of the community structure in a given partition by evaluating how much more communities are connected than expected by a random background model. The two methods which we use differ in the choice of this background model. Modularity Maximization partitions the network in such a way as to maximize this measure.

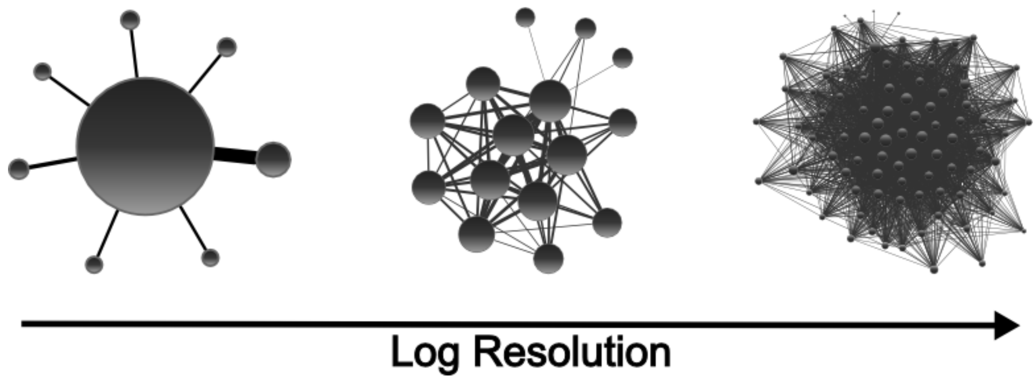

Fig S.1: Diagram showing the effect of the resolution parameter in community detection. The circles represent communities and the lines denote edges between them. The size of a circle, or a line, is proportional to the community size, or the number of edges between two communities, on a logarithmic scale. The three graph representations show network partitions generated at different resolutions by the configuration model Modularity Maximization community detection method implemented on HINT-P. Different community decompositions are generated at different resolutions.

Here, we use the Modularity adaptation proposed by (Reichardt and Bornholdt, 2006) which includes a resolution parameter, γ. For a network on N nodes, modularity, H_γ, is calculated by the function:

$$H_\gamma(\sigma_i, \ldots, \sigma_N) = \sum_{i,j}(A_{ij} - \gamma p_{ij})\delta(\sigma_i, \sigma_j). \tag{1}$$

In this equation, σ_i denotes the community membership of node i, A_{ij} denotes the adjacency matrix of the network, and p_{ij} denotes the probability of interaction between nodes i and j under the background model used. As indicated by the Kronecker-Delta function $\delta(\sigma_i, \sigma_j)$, which is 1 if $\sigma_i = \sigma_j$ and 0 otherwise, the sum is taken over all nodes i and j which are in the same community. The interaction strength, $A_{ij} - \gamma p_{ij}$, gives an indication of how well connected the two nodes i and j are, compared to what is expected under the background interaction model, p_{ij}.

The null, or background, model should thus summarize what we expect our protein interaction network to look like. Pairs of nodes that are connected more than expected from this null model and the resolution factor, γ, increase the Modularity function and are thus aimed to be assigned to the same community by the algorithm. The lower the value of γ, the more likely it is that two nodes are assigned to the same community and larger communities will be created. This interplay of resolution parameter and null model shows that the choice of null model is central to the network partition obtained from multi-scale Modularity optimization. The two null models used here are given by the equations:

$$p_{ij} = \frac{k_i k_j}{2m}, \quad \text{for}\, i \neq j, \tag{2}$$

for the configuration model, and

$$p_{ij} = p = 1, \quad \text{for}\, i \neq j, \tag{3}$$

for the Constant Potts model. Here k_i denotes the degree of node i, and m is the total number of edges in the network as calculated by $\sum_{ij} A_{ij}/2$. While the configuration model is a commonly used null model, which conserves the degree distribution of the network (Lewis *et al.*, 2010; Reichardt and Bornholdt, 2006), the Constant Potts model proposed by (Traag *et al.*, 2011) compares the PIN to a complete graph with all possible edges present (Bernoulli

random graph). These two null models describe the expected network very differently and therefore produce different network partitions.

The overlapping community detection methods used here represent different types of overlap. Link clustering assumes sparse overlaps between communities, while BigClam models overlaps between communities as dense regions of interactions.

The BigCLAM method (Yang and Leskovec, 2013) (Cluster Affiliation Model for big networks) describes the network partition into communities as a bipartite graph between nodes and communities. Here, the number of communities, K, is assumed given. Each edge in this graph represents a non-zero "interaction strength", F_{uA}, of a node u in a community, A, (Yang and Leskovec, 2013). This interaction strength parametrizes how strongly node u is connected with other nodes in the community A. Each node, u, is thus assigned a $1 \times K$ vector of interaction strengths, F_u. The probability that two nodes u and v interact, $p(u,v)|_{(u,v)\in E}$, scales with the number of community assignments that u and v share, and is chosen to be:

$$p(u,v)|_{(u,v)\in E} = 1 - \exp(-F_u \cdot F_v^T).$$

Here, F_v^T is the transpose of the vector of interaction strengths of node v. The optimal bipartite graph representing the community decomposition is found by determining the values of F which, given the graph G, maximize the log likelihood function, $l(F) = \log P(G|F)$ (Yang and Leskovec, 2013):

$$l(F) = \sum_{(u,v)\in E} \log(1 - \exp(-F_u F_v^T)) - \sum_{(u,v)\notin E} F_u F_v^T.$$

Here, $(u,v) \in E$ denotes all node pairs connected by an edge in the edge set E of the graph. The individual interaction strengths, F_{uA}, are translated into binary community memberships by a thresholding effect. When F_{uA} is greater than a threshold, usually set to the background interaction probability in the PIN (the density), the node is regarded as affiliated with community A.

A second method for overlapping community structure particularly suitable for sparse overlaps is Link clustering (Ahn *et al.*, 2010). Link clustering is an edge-based clustering technique which computes the similarities of edges that share a so-called "keystone node", k, by:

$$s(e_{ik}, e_{jk}) = \frac{|n_+(i) \cap n_+(j)|}{|n_+(i) \cup n_+(j)|}. \tag{4}$$

Here e_{ik} denotes the edge between nodes i and k, and $n_+(i)$ is the inclusive neighbour set of node i, defined as the set of neighbours of node i including the node itself. All edges with a similarity value above a threshold, S, are clustered together to form edge communities, where $S \in [0,1]$ is the resolution parameter. Thus, for $S = 0$ there is only one community. Nodes are assigned to all the communities their edges are members of so that each node can be in as many communities as it has edges.

1.2 Semantic Similarity

Semantic similarity measures quantify the similarity of two objects based on a structured ontology of terms that are used to describe the objects. The relationships between the terms associated with each object is used to calculate their similarity. To quantify protein functional similarity, the preferred structural ontology is the biological process (BP) part of the gene ontology (GO) (Ashburner *et al.*, 2000).

Following reviews of semantic similarity measures in PINs and other biological applications (Guzzi *et al.*, 2012; Mazandu and Mulder, 2014; Pesquita *et al.*, 2009), we chose three semantic

similarity measures: simUI (Gentleman, 2005), simGIC (Pesquita *et al.*, 2008), and the Pandey measure (Pandey *et al.*, 2008).

The simUI measure (Gentleman, 2005) uses the Jaccard index between the GO-term sets, S_i and S_j, of two proteins, i and j, to compute their similarity by the equation:

$$\rho(S_i, S_j) = \frac{|S_i \cap S_j|}{|S_i \cup S_j|}. \tag{5}$$

The simGIC semantic similarity measure (Pesquita *et al.*, 2008) is an extension of simUI, which uses the cumulative information content of the GO-term sets instead of their cardinality. The information content of a GO-term is calculated by $IC = -\log_2(p)$, where p is the probability of finding this term associated with a randomly picked protein in an external database, such as the PIN itself.

The Pandey measure (Pandey *et al.*, 2008) implements an approach similar to information content extended to GO-term sets. Proteins whose intersubsection sets are common among proteins in the PIN are judged to be less similar than those with rare intersubsection sets. The equation for this similarity measure is:

$$\rho(S_i, S_j) = -\log_2 \left(\frac{G_{\Lambda(S_i,S_j)}}{G_r} \right). \tag{6}$$

Here, $G_{\Lambda(S_i,S_j)}$ denotes the number of proteins in the PIN which are annotated with the intersubsection set of the GO-term sets S_i and S_j of proteins i and j respectively, and G_r represents the total number of annotated proteins in the network.

To show how these semantic similarity measures differ in a practical application, we display the distribution of similarity scores for proteins in HINT-P using the three measures (Figure S.2).

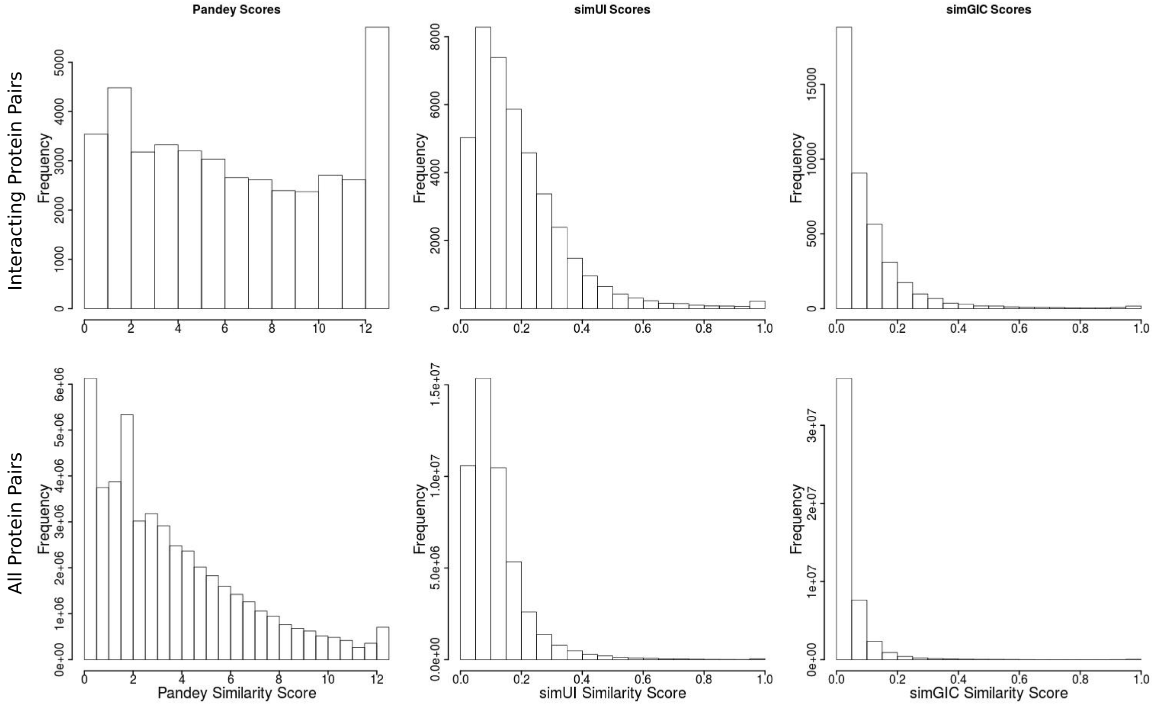

Fig S.2: Distribution of semantic similarity scores for the Pandey measure, simUI, and simGIC between interacting protein pairs (top row), and all protein pairs (bottom row) in HINT-P. The scores clearly differ for interacting proteins compared to all proteins, with Pandey scores showing the strongest signal. Furthermore, the interacting protein score distributions show that the median score will be far lower than the mean score for simGIC.

We use these semantic similarity measures to compute the functional homogeneity of a group of proteins by averaging the similarity scores of all protein pairs in the community. Proteins without GO BP annotations are omitted in this calculation.

2 Appendix B: Inspection Bias Results

We investigated how research focus affects module evaluation in two protein interaction networks (HINT-P and BioGrid-AP, cf. *Methods*). To perform this investigation, it is important to quantify how well-studied a protein is, and how similar a protein is judged to be to others. Following reviews of functional similarity measures (Guzzi *et al.*, 2012; Mazandu and Mulder, 2014; Pesquita *et al.*, 2009), we chose simUI (Gentleman, 2005), simGIC (Pesquita *et al.*, 2008), the Pandey measure (Pandey *et al.*, 2008), and functional enrichment (Huang *et al.*, 2009) to represent different approaches to quantify the functional homogeneity of a set of proteins. These sets of proteins were obtained by performing 10,000 random walks of a certain size from each node in the tested PINs. Using random walks to represent proxy modules in this way, the effect of research focus on module evaluation was evaluated independently of any specific community detection method.

We quantified how well-studied a proxy module is based on the fraction of proteins that are annotated in each module (annotation fraction). To ensure we can obtain more than six unique annotation fraction values for length six random walks, all random walks (proxy modules) started from the same node were averaged. We thus perform the correlation test on node "vicinities" defined by all proxy modules centred on a node, rather than the individual proxy modules.

The functional homogeneity of these node vicinities were also computed via the random walk proxy modules and averaged. Using the three semantic similarity measures, the functional homogeneity of the random walks was calculated by taking the average of the pairwise semantic similarities of all annotated proteins. Functional enrichment using the hypergeometric distribution was used to calculate functional homogeneity by taking the p-value of the most enriched GO term. To ensure that the p-values are comparable between modules and allow for averaging of the functional enrichment functional homogeneity scores in a node vicinity, two steps were taken. Firstly, we did not correct for multiple testing and secondly, random walk lengths were determined based on annotated nodes instead of traversed nodes. By omitting multiple-testing correction the p-values were not scaled by the number of terms annotated to proteins in a proxy module. While this prevents the p-value from being used as an evaluation of the statistical significance of a module, it instead allows us to treat it as an enrichment score. The length of random walks was determined by annotated nodes instead of traversed nodes such that the range of enrichment scores for proxy modules were equal. If two proxy modules both contain six nodes, however only five of these are annotated in one of the modules, then the maximum number of nodes that can share an annotation differs in these proxy modules (five and six depending on the number of annotated nodes). Thus, the minimum possible enrichment scores also differ. By terminating the random walks only after traversing a predetermined number of functionally annotated nodes, equal sample sizes are drawn from the set of annotated nodes, making enrichment scores comparable.

The node vicinity functional homogeneity and annotation fraction scores were ranked, and the Pearson correlation coefficient between the ranked variables was calculated - the so-called Spearman correlation coefficient. Under the null hypothesis of independence, the standard error of the Spearman correlation coefficient is $\frac{0.67449}{\sqrt{(n-1)}}$ (Pearson, 1907), where n is the number of nodes (0.006 for HINT-P and 0.005 for BioGrid-AP). The Spearman's correlation coefficients for the node vicinity annotation fraction and functional homogeneity scores on HINT-P and

BioGrid-AP are shown in Table S.1. All of the scores are significantly different from 0, the uncorrelated case. The lower correlation scores for the BioGrid-AP network are likely due to a higher false positive rate in this network.

Table S.1: Research focus random walk investigation

Network	Functional Similarity	Walk Length 3	Walk Length 6
HINT-P	Pandey	0.414	0.572
	SimUI	0.200	0.202
	SimGIC	0.240	0.269
	Func Enrich	-0.473	-0.621
BioGrid-AP	Pandey	0.209	0.266
	SimUI	0.107	0.085
	SimGIC	0.134	0.104
	Func Enrich	-0.354	-0.409

Spearman correlation coefficients between the functional similarity scores and the fraction of annotated proteins in random walks are quoted for four functional similarity measures. Note that negative correlations are expected for functional enrichment, as more functionally homogeneous proteins exhibit lower p-value scores in contrast to the semantic similarity measures. All correlations are significantly different from zero, based on a standard normal approximation.

The correlation of these quantities shows that proteins with less functionally annotated proteins in their vicinity (poorly studied vicinities) tend to also have a lower functional similarity to proteins in their vicinity. Given that the correlation is based on protein vicinities, we conclude that there are regions in PINs with fewer annotations and low functional similarities, and those with more annotations and high functional similarities.

3 Appendix C: CommWalker Implementation Details

CommWalker is a method framework designed to counteract the effect of research focus on module evaluation. It performs random walks to sample the local network environment of communities in order to relate the similarity scores of nodes within these communities to the similarity scores of nodes around them. Using this comparison CommWalker assigns a significance score to the communities (cf. Figure S.3a). Given a semantic similarity measure

and a network partition, the CommWalker algorithm is implemented as follows:

Algorithm 3.1: CommWalker(partition, FH())

```
comment: W_N - Number of random walks started from each node in the community
comment: FH() - Semantic similarity measure to compute the functional homogeneity
comment: f() - function to compute W_N based on the community size (explained later)
for each comm ∈ partition
  do {
    W_N ← f(|comm|)
    commFH ← FH(comm)

    M ← 0
    m ← 0

    for each node ∈ comm
      do {
        for i ← 1 to W_N
          do {
            random walk from node
            randFH ← FH(random walk)
            M ← M + 1

            if (randFH > commFH)
              then m ← m + 1
          }
      }

    T-value ← (m + 1)/(M + 1)
  }
```

The stability of the significance score output by this algorithm depends on how well the local network environment was captured in the sampling process. To investigate when the environments of all communities are extracted adequately, we quantified the resampling of a community via the node score, Z, as follows.

For a community, C, of size N_C, each random walk started from the community visits N_C distinct nodes. If $W_N(C)$ random walks are started at each node in the community, a total of $Z(C) = W_N(C)N_C^2$ nodes are sampled by the random walks from community C. We call $Z(C)$ the node count for community C. To compare the amount of resampling between communities we keep the node count, $Z(C)$, near constant. Thus, the number of random walks per node for a community C (denoted f(commSize) in the pseudocode above), can be calculated by the equation:

$$W_N(C) = \frac{Z}{N_C^2}. \tag{7}$$

We round $W_N(C)$ up to the next integer so that the same number of random walks can be started at each node in the community, hence preventing a background sampling bias. Due to this rounding the actual number of nodes visited in the completed random walks from each community will tend to be slightly higher than Z. As a consequence of keeping Z near constant for all communities, more random walks are performed for smaller communities, as each random walk is shorter. Alternative ways to scale the number of random walks with community size were investigated and found to lead to T-value stability depending on community size.

To find what value of the node count, Z, gave the best trade-off between T-value stability and algorithm run-time, we randomly selected nine communities of sizes ≤ 35 from the config-

uration model Modularity Maximization HINT-P data set for repeated T-value measurement (Fig. S.3b). The stability of the T-value of a community was determined by running 100 repeats of CommWalker on the same community and taking the standard error of the resulting T-value samples.

The stability of the nine randomly selected communities was calculated at five different node counts. Figure S.3b shows the trade-off between T-value stability and the node count, which is linearly related to the run time of the algorithm. Given this data a node count of 100,000 was selected. As estimated based on the nine communities tested, T-values are taken with an associated standard error of ≈ 0.005 at this node count. The stability calculation is shown here for a sample of HINT-P communities. T-value stability investigation on BioGrid-AP (data not shown) produced similar results. T-value stability on other networks may vary depending on the network structure.

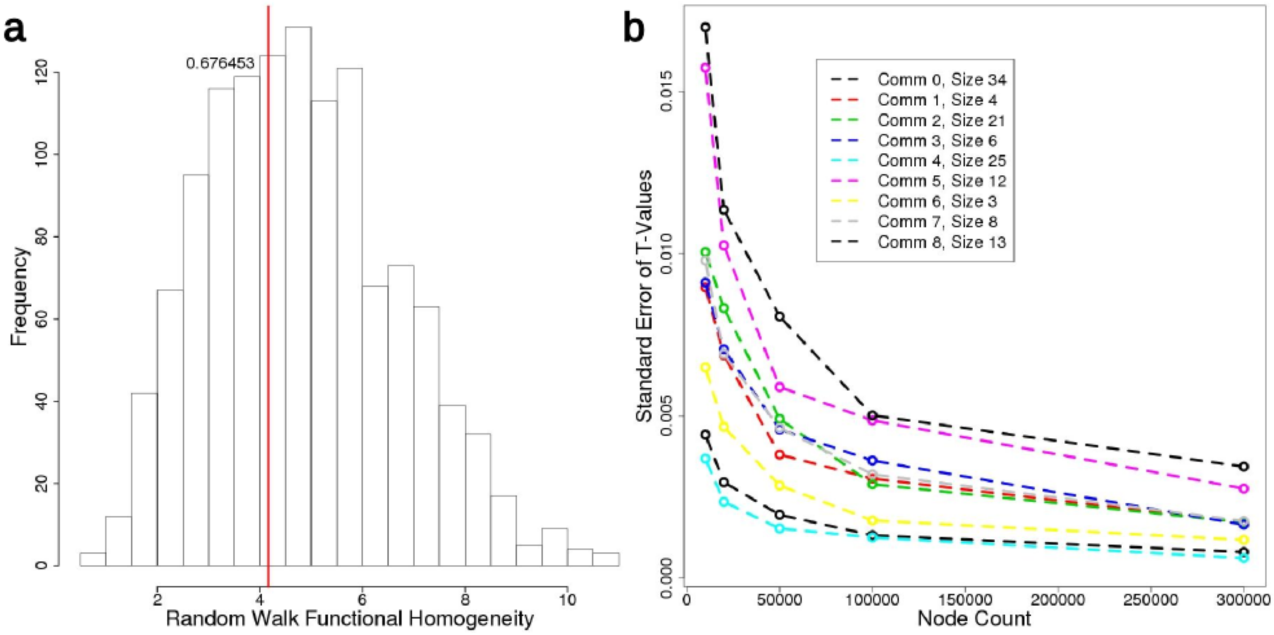

Fig S.3: T-value calculation and stability. (a) Background functional homogeneity distribution generated from 2,500 random walks for a community of size 6 in HINT-P. The red vertical line represents the community's functional homogeneity and its associated T-value. (b) Standard errors of community T-values computed over 100 CommWalker runs on nine randomly selected communities of sizes ≤ 35 from the configuration model Modularity Maximization HINT-P data set. The number of random walks started at each node is calculated from the node count by equation 7. The trade off between the stability of T-values and the run time, which is proportional to the node count, is adjudged optimal at a node count of 100,000 based on this HINT-P sample. The random walk functional homogeneity distribution for the community denoted "Comm 3" at a node count of 10,000 is shown in (a).

Based on the T-value stability investigation, the node count was set at a value that optimizes the trade-off between T-value stability and run time. To further ensure a fast run time for CommWalker and a high stability of computed T-values, we implemented two filters in the algorithm. The first filter is the community size that can be evaluated. A hard lower boundary for community size was set at three proteins, which is the minimum non-trivial community size. An upper boundary on the community size was set by the heuristic equation $\frac{X}{15} + 20$, where X is the number of nodes in the PIN. With currently available PINs often containing $> 10,000$ proteins, this boundary filters out communities that are unlikely to be of biological interest and are slow to evaluate.

The second filter was implemented on the random walks. Random walks can fail for two reasons. Firstly, the walk may become trapped in a bottleneck in the network, and secondly it

may be that no two proteins in a completed walk can be compared due to a lack of annotation. The first case is overcome by allowing a maximum of N_C^2 steps when attempting to reach N_C nodes, where N_C is the number of nodes in the community. For the second case we further imposed a maximum number of random walk attempts. CommWalker aims to perform $W_N(C) \times N_C$ successful random walks per community (cf. Equation 7), where the total number of attempts allowed to reach this target is twice this number. The number of times that the random walks fail per community is output in a separate file by the software.

The code is available at `http://www.stats.ox.ac.uk/research/proteins/resources`.

4 Appendix D: Module Analysis Results

4.1 Coverage of accepted modules

To investigate the efficacy of the CommWalker framework, we compare the communities that are positively evaluated by CommWalker with those that are positively evaluated by functional homogeneity without CommWalker. Here, positive evaluation by CommWalker refers to those communities whose T-value is below the threshold of 0.5, and positive evaluation by functional homogeneity refers to communities whose functional homogeneity value is above the median semantic similarity of interacting proteins (Med int BG). These acceptance thresholds were chosen as they are qualitatively similar.

Using these thresholds we compared the number of unique proteins in communities that are accepted by CommWalker, and functional homogeneity without CommWalker, using the Pandey measure (Figures S.4–S.7), simGIC (Figures S.8 and S.9), and simUI (Figures S.10 and S.11). Using simUI and the Pandey measure there are consistently more unique proteins in CommWalker accepted communities than in those accepted by functional homogeneity. Using simGIC, this number tends to be more similar. This observation may be explained by the simGIC score distribution (Figure S.2), which is more skewed than those of simUI and the Pandey measure. In this case the median of the similarity scores is further below the mean value than in the other cases, resulting in the functional homogeneity threshold being more lenient.

4.2 Module prioritization

The investigation in the Appendix D Section 4.1 above shows that for qualitatively similar thresholds, CommWalker accepts more communities than functional homogeneity. This result prompts the question whether more communities being functionally significant is a byproduct of differences in the qualitatively similar thresholds, or is due to the intended reprioritization of modules. To address this question, we computed the overlap between the communities evaluated as functionally significant by CommWalker and functional homogeneity.

For each data set we chose a network partition generated at a single resolution. This resolution was chosen to maximize the proteins in functionally significant communities at a T-value threshold of 0.5 (see maxima in Figures S.4–S.7). For a given T-value threshold the set of proteins in T-value-significant communities at this resolution was computed, and an equivalent functional homogeneity threshold was identified which gave the most similar number of proteins in functional-homogeneity-significant communities. The overlap of these two protein sets was calculated to determine the similarity of the two evaluation approaches. This investigation was repeated for both PINs, the four community detection methods, and several T-value thresholds with Pandey semantic similarity (Table S.2). We found that while there was an overlap between the two evaluation methods, the ordering of communities according to T-value was inherently different to that of functional homogeneity irrespective of T-value threshold.

PIN	Community Detection	T ≤ 0.05	T ≤ 0.1	T ≤ 0.25	T ≤ 0.5
HINT-P	Link	0.631	0.724	0.839	0.948
	BigCLAM	0.630	0.791	0.873	0.964
	Config Mod	0.677	0.663	0.737	0.891
	CPM Mod	0.547	0.620	0.776	0.925
BioGrid-AP	Link	0.758	0.816	0.896	0.950
	BigCLAM	0.713	0.795	0.907	0.984
	Config Mod	0.730	0.761	0.848	0.950
	CPM Mod	0.648	0.727	0.816	0.933

Table S.2: The fraction of proteins that are common to both the top T-value ranked communities and the top functional homogeneity ranked communities for all PIN and community detection method combinations using Pandey semantic similarity. The set of unique proteins in communities with a T-value below the given thresholds is compared to the set of proteins in the highest ranked functional homogeneity communities of the most similar size. The fraction is calculated by dividing by the smaller protein set.

The overlap between the two protein sets at higher thresholds shows that CommWalker confirms a large fraction of the communities found by functional homogeneity. The communities evaluated as functionally significant by both methods are prime candidates for functional modules or protein complexes as they are verified by a two-pronged approach. These communities tend to contain well-studied proteins (see Figure 3(b) in the paper).

In contrast, especially at low T-value thresholds, the T-value and functional homogeneity protein sets are different. At a T-value threshold of 0.05, overlaps for the different networks and methods range from ≈55% to ≈76%. The different selection of communities by T-value and functional homogeneity evident from these results shows that CommWalker's approach of evaluating communities based on whether they stand out from their environment prioritizes different communities from classical evaluation methods. CommWalker not only identifies more functionally significant communities than evaluation based solely on functional homogeneity, but it also evaluates them in a different way and therefore represents a previously unconsidered source of insight.

4.3 Module Statistics

To assess whether modules accepted by CommWalker succeed in counteracting the inspection bias effects we describe in the S2 Appendix, we evaluated the characteristics of those communities which are adjudged significant by one method but not the other. Using the qualitatively similar thresholds mentioned previously, the communities were divided into four sets: accepted by both, rejected by both, accepted by only CommWalker, and accepted by only functional homogeneity. Average network statistics for these community sets were calculated for both HINT-P and BioGrid-AP, all four community detection methods, and all three semantic similarity measures. The results are shown in Figures S.12–S.17.

The four community sets were analyzed for their average community sizes, the average level of annotation in their local environments (average random walk annotation fraction), and the average level of annotation of the communities (average community annotation fraction). The upper row of graphs in Figures S.12–S.17 show that communities accepted only by functional homogeneity are the smallest set for the Pandey measure and simUI, and communities accepted only by CommWalker are the smallest set using simGIC. In some data sets the smallest set become so small that the summary statistics on it are very variable, which affects our ability to interpret them. This difficulty can be observed in both Pandey measure Modularity maximization data set analyses of the annotation levels in the BioGrid-AP data (bottom right quadrant Figure S.12). The grey lines here are very variable due to a low sample size. Towards

higher resolutions, where the fraction of communities only accepted by functional homogeneity increases, the plots become steadier and therefore more reliable to interpret. The same effect occurs in the BioGrid-AP BigCLAM annotation fraction graphs. Until a number of fitted communities of 2,251 there is only a single community in the set of communities accepted by only functional homogeneity (grey line). A further jump in the grey lines can be seen at 2,751 fitted communities, when the number of communities jumps from three to seven. The small sample size is specifically an issue for the simGIC data sets in Figures S.14 and S.15, and the simUI BioGrid-AP data set in Figure S.16.

Taking small sample size effects into account, Figures S.12–S.17 show that using functional homogeneity thresholding selects for smaller community sizes, for communities that have a higher proportion of annotated nodes, and for those that are in well-annotated environments.

As we have no reason to believe that biologically relevant communities should be in certain ranges of the investigated statistics, it is advantageous if the communities that are evaluated as significant by a specific method show a broad distribution of these statistics. This effect can be seen for the communities accepted only by CommWalker in all investigated statistics, but especially the average random walk annotation fraction. Here, the blue lines tend to be more similar to the turquoise dashed lines for communities mutually rejected, than the red dashed lines for communities mutually accepted. Hence both lines representing communities accepted by CommWalker (black dashed line and blue line) are in different ranges of average random walk comparison fraction. As the turquoise line represents ranges of these statistics in which significant communities are difficult to detect, the results show that CommWalker can find communities that are significant even in ranges of the network annotation statistic in which communities tend to be rejected. Hence, CommWalker may detect functionally significant communities in regions of the network otherwise obscured by poor annotation.

This conclusion is further supported by Figure 3 in the paper, and Figures S.18–S.28. In these figures, non-overlapping community data generated by configuration model and Constant Potts model Modularity Maximization on HINT-P and BioGrid-AP was used to show the distribution of proteins in accepted modules on the PINs for the three semantic similarity measures. In Figures S.18–S.28 the proteins are ordered by their semantic similarity with their "vicinity", measured using random walks as described in the S2 Appendix. Proteins towards the left have higher similarity with their environment and will thus tend to be better studied. On this layout we show the distribution of proteins in communities that were accepted as modules by both methods (row b), only by CommWalker (row c), or only by functional homogeneity (row d). Figures S.18–S.28 show that across all data sets proteins in modules accepted by the standard functional homogeneity approach (rows b,d) tend to be distributed towards the well-studied left side of the figure. In contrast, modules accepted only by CommWalker (row c) reach further into the poorly-studied protein regions.

Due to ease of visualization only non-overlapping community data was used for this investigation, however judging from the module statistics displayed in Figures S.12–S.17 it can be concluded that CommWalker is equally successful in the overlapping case.

HINT-P Pandey

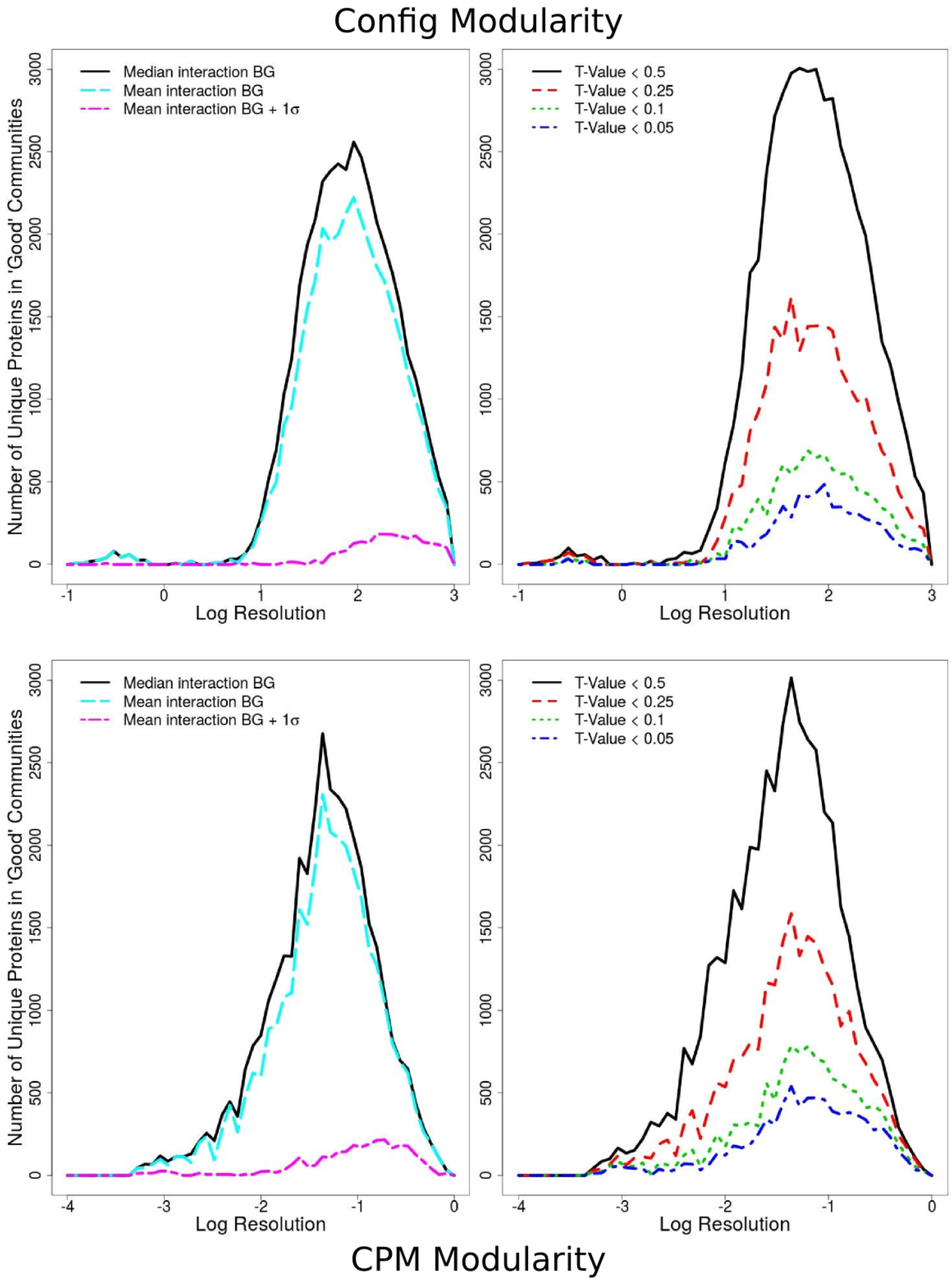

Fig S.4: Comparison between the number of unique proteins judged to be in functionally significant communities of size 6 - 35 by functional homogeneity (left hand plots), and T-value thresholds (right hand plots) using the Pandey measure. The two cases can be compared for the qualitatively similar thresholds of a T-value of 0.5 and the median functional similarity of interacting proteins (Median Interaction BG – black, solid lines). Communities were generated by the Configuration model Modularity Maximization and Constant Potts model Modularity Maximization community detection methods applied to HINT-P. The number of unique proteins in T-value-significant communities is consistently higher than in functional-homogeneity-significant communities for qualitatively similar thresholds. Using the mean functional similarity of interacting proteins (Mean interaction BG), the mean interaction BG with an added standard error of the interacting proteins' functional similarity scores (Mean interaction BG $+1\sigma$), and different T-values, we further highlight the number of unique proteins at different T-value and functional homogeneity thresholds.

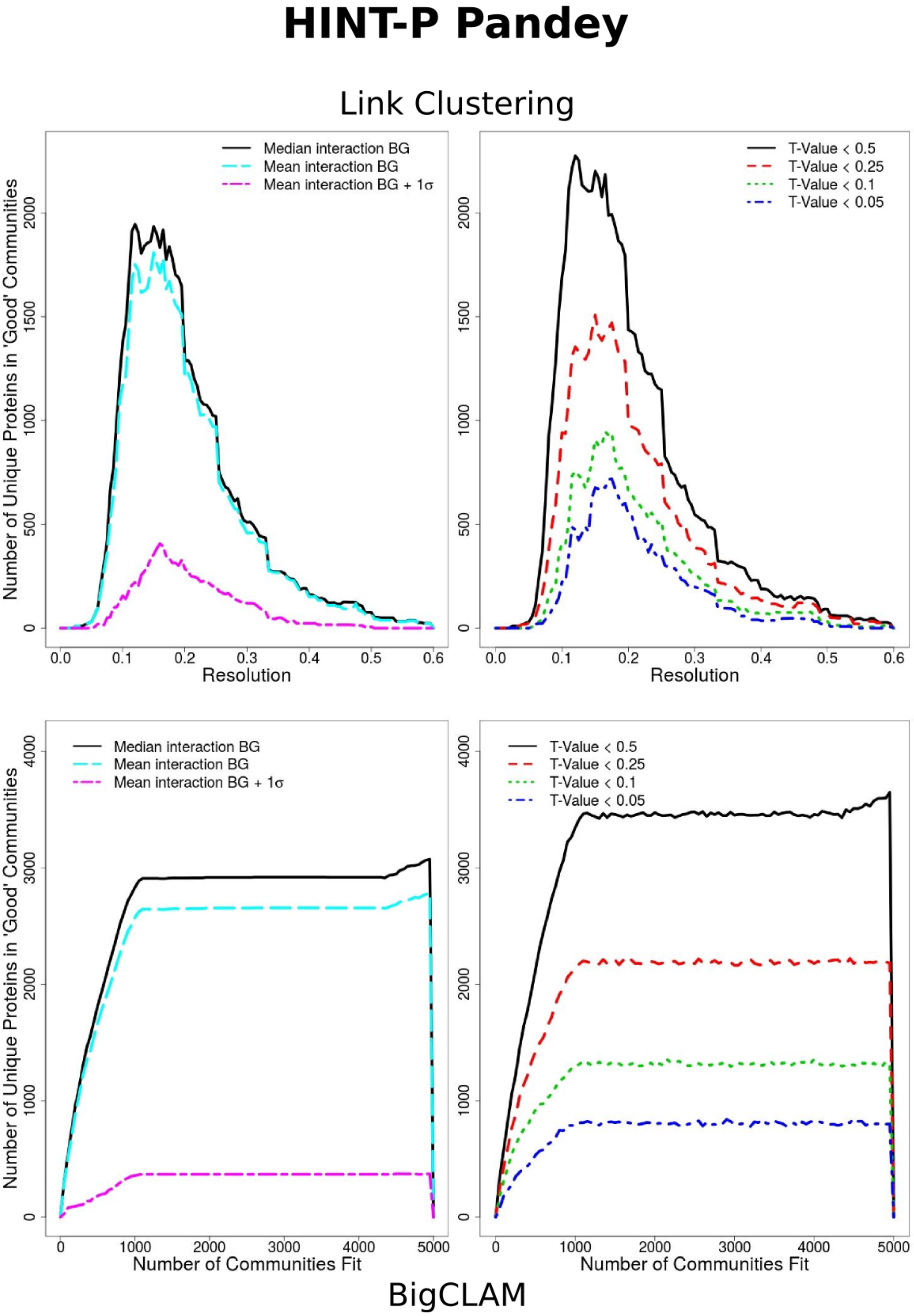

Fig S.5: Comparison between the number of unique proteins judged to be in functionally significant communities of size 6 - 35 by functional homogeneity (left hand plots), and T-value thresholds (right hand plots) using the Pandey measure. The two cases can be compared for the qualitatively similar thresholds of a T-value of 0.5 and the median functional similarity of interacting proteins (Median Interaction BG – black, solid lines). Communities were generated by the Link clustering and BigCLAM community detection methods applied to HINT-P. The number of unique proteins in T-value-significant communities is consistently higher than in functional-homogeneity-significant communities for qualitatively similar thresholds. Using the mean functional similarity of interacting proteins (Mean interaction BG), the mean interaction BG with an added standard error of the interacting proteins' functional similarity scores (Mean interaction BG $+1\sigma$), and different T-values, we further highlight the number of unique proteins at different T-value and functional homogeneity thresholds.

BioGrid-AP Pandey

Config Modularity

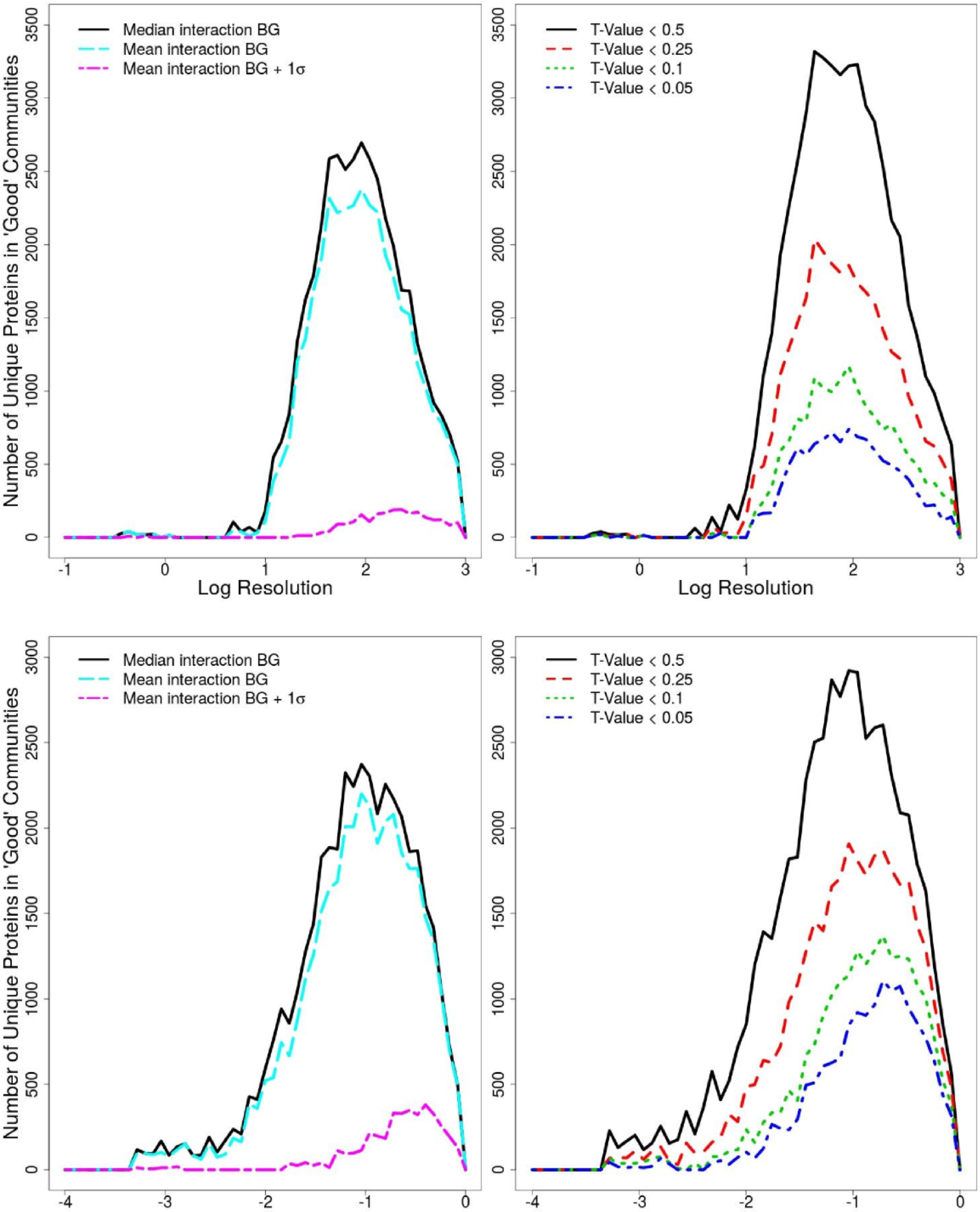

CPM Modularity

Fig S.6: Comparison of the number of unique proteins in significant communities of size 6 - 35 by T-value and functional homogeneity. The data was generated as in Figure S.4 using the Pandey measure with BioGrid-AP. The number of unique proteins in T-value-significant communities is consistently higher than in functional-homogeneity-significant communities for the qualitatively similar thresholds indicated by the black lines. Using the mean functional similarity of interacting proteins (Mean interaction BG), the mean interaction BG with an added standard error of the interacting proteins' functional similarity scores (Mean interaction BG $+1\sigma$), and different T-values, we further highlight the number of unique proteins at different T-value and functional homogeneity thresholds.

BioGrid-AP Pandey

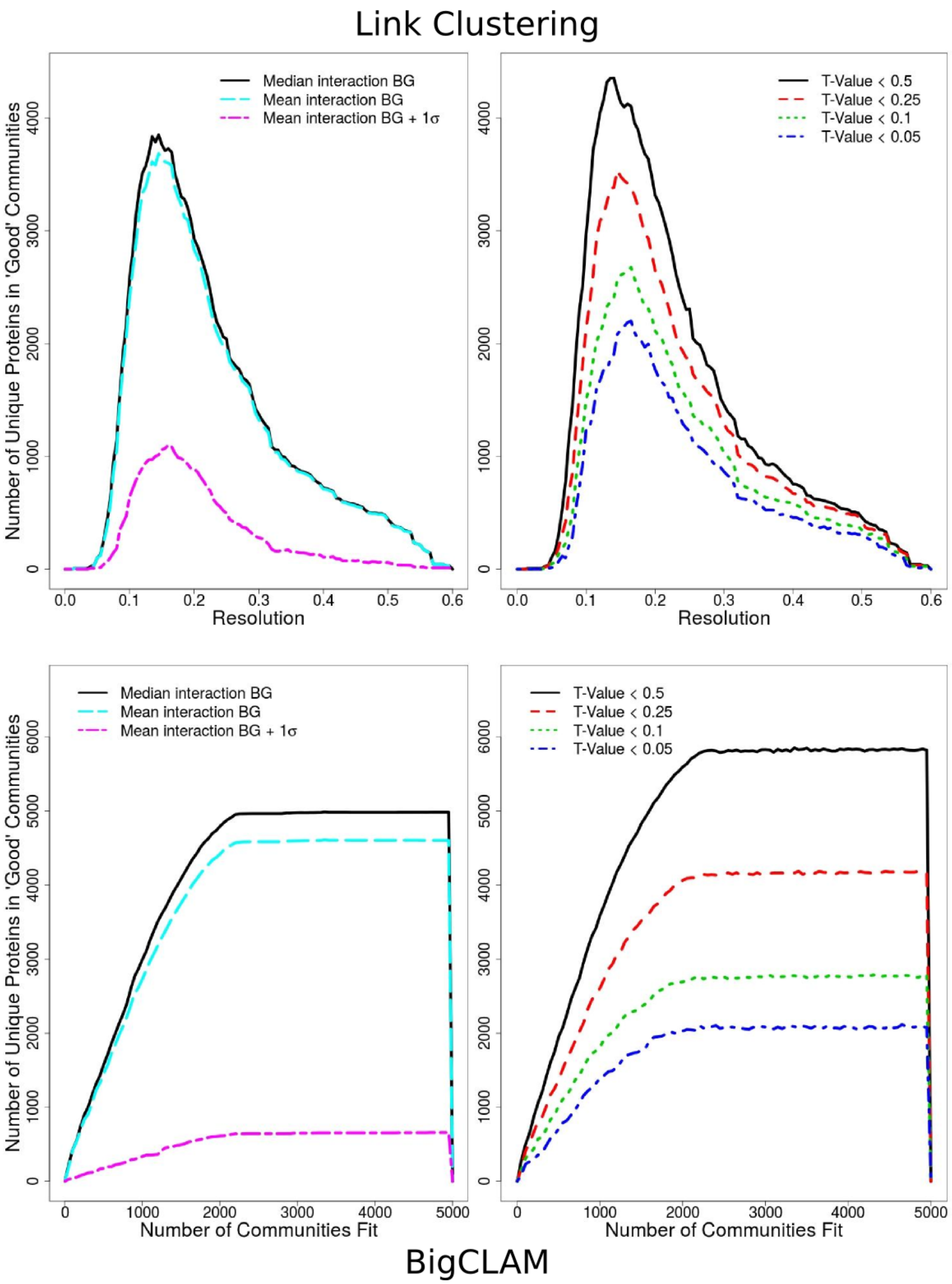

Fig S.7: Comparison of the number of unique proteins in significant communities of size 6 - 35 by T-value and functional homogeneity. The data was generated as in Figure S.5 using the Pandey measure with BioGrid-AP. The number of unique proteins in T-value-significant communities is consistently higher than in functional-homogeneity-significant communities for the qualitatively similar thresholds indicated by the black lines. Using the mean functional similarity of interacting proteins (Mean interaction BG), the mean interaction BG with an added standard error of the interacting proteins' functional similarity scores (Mean interaction BG $+1\sigma$), and different T-values, we further highlight the number of unique proteins at different T-value and functional homogeneity thresholds.

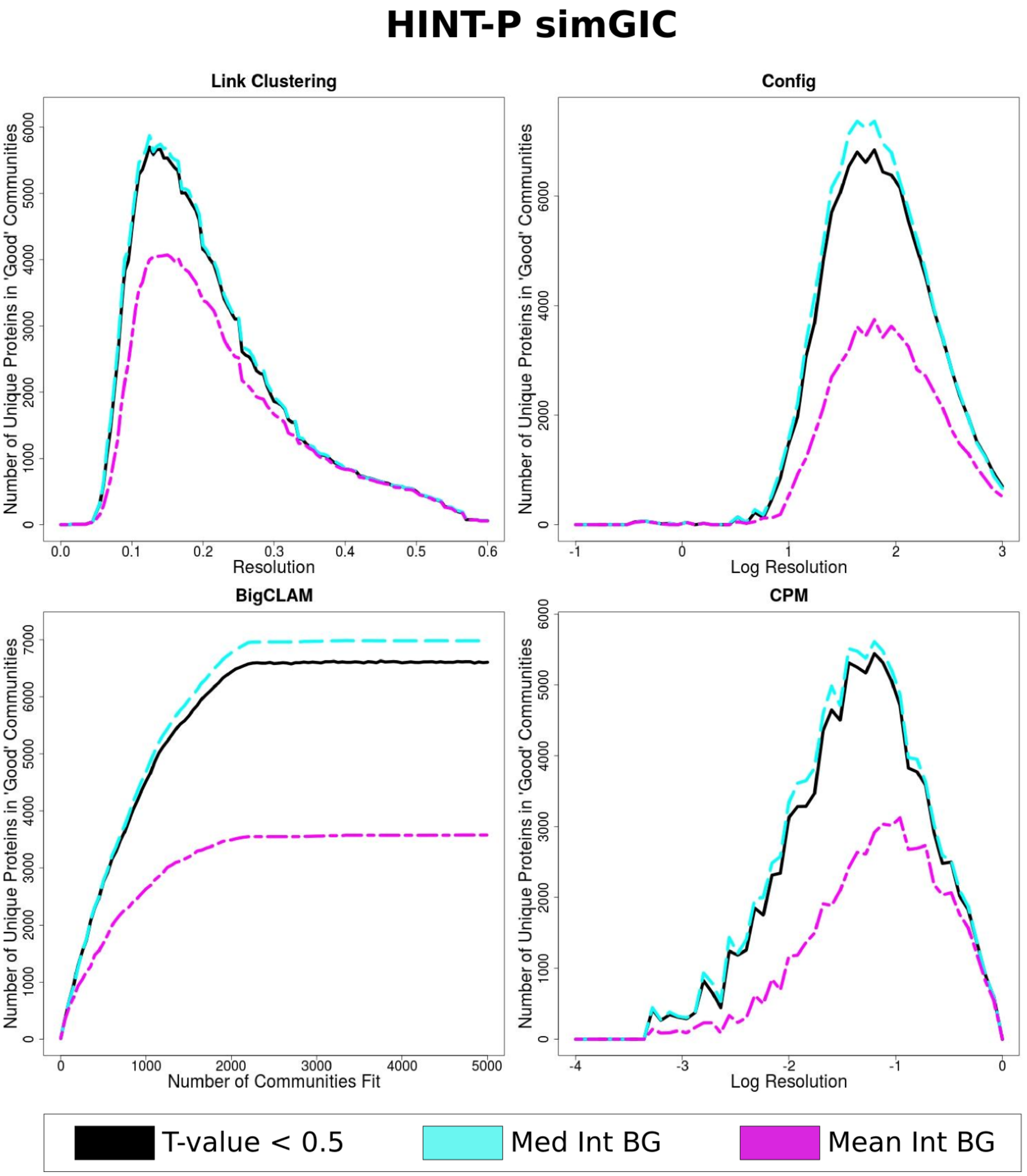

Fig S.8: Comparison of the number of unique proteins in significant communities of size 6 - 35 by T-value and functional homogeneity. The data was generated as in Figures S.4 and S.5 using the simGIC semantic similarity measure on HINT-P. Using simGIC, the number of unique proteins in T-value-significant communities tends to be similar to the number in functional-homogeneity-significant communities for the qualitatively similar thresholds indicated by the black and cyan lines.

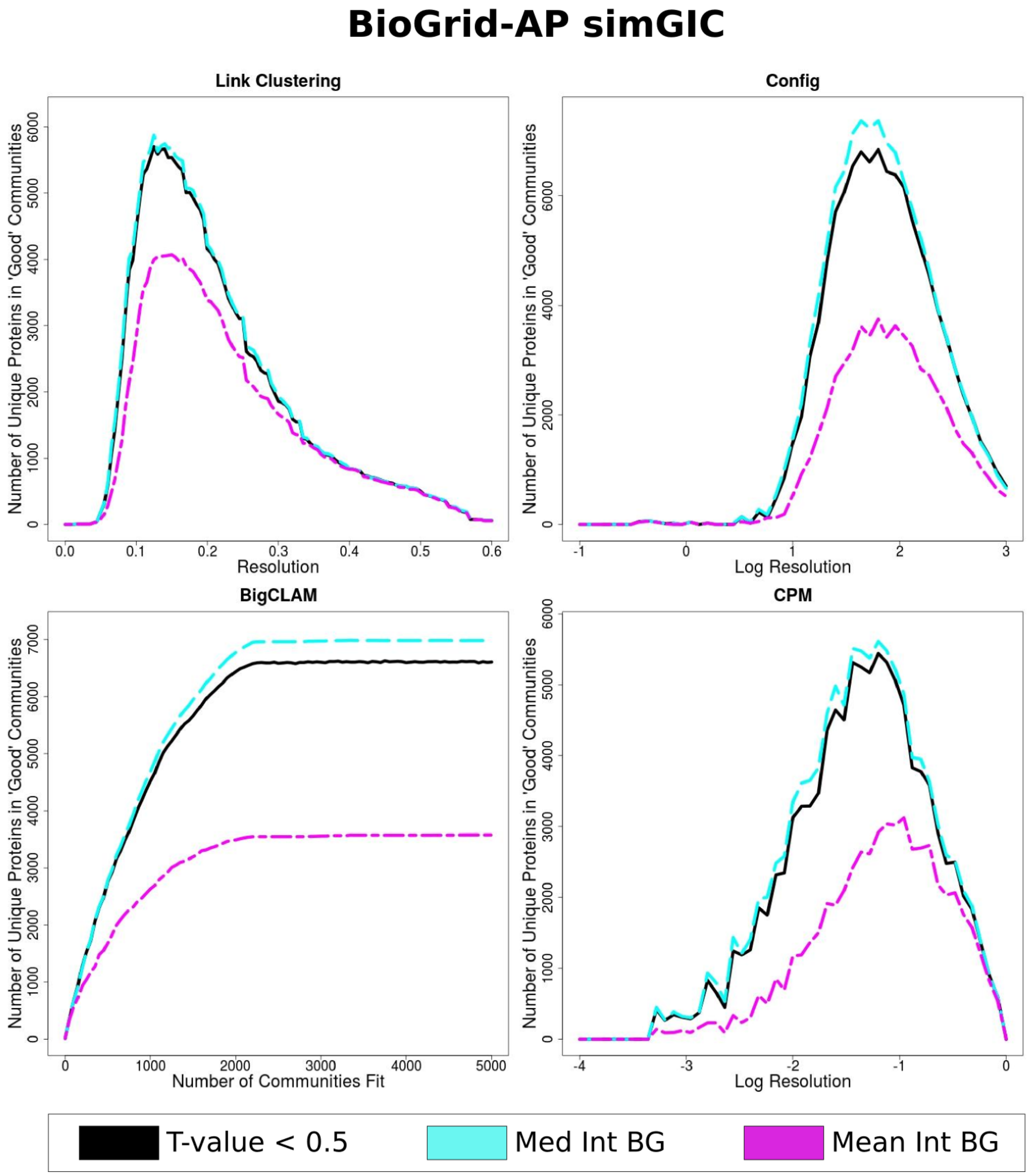

Fig S.9: Comparison of the number of unique proteins in significant communities of size 6 - 35 by T-value and functional homogeneity. The data was generated as in Figures S.4 and S.5 using the simGIC semantic similarity measure on BioGrid-AP. Using simGIC, the number of unique proteins in T-value-significant communities tends to be similar to the number in functional-homogeneity-significant communities for the qualitatively similar thresholds indicated by the black and cyan lines.

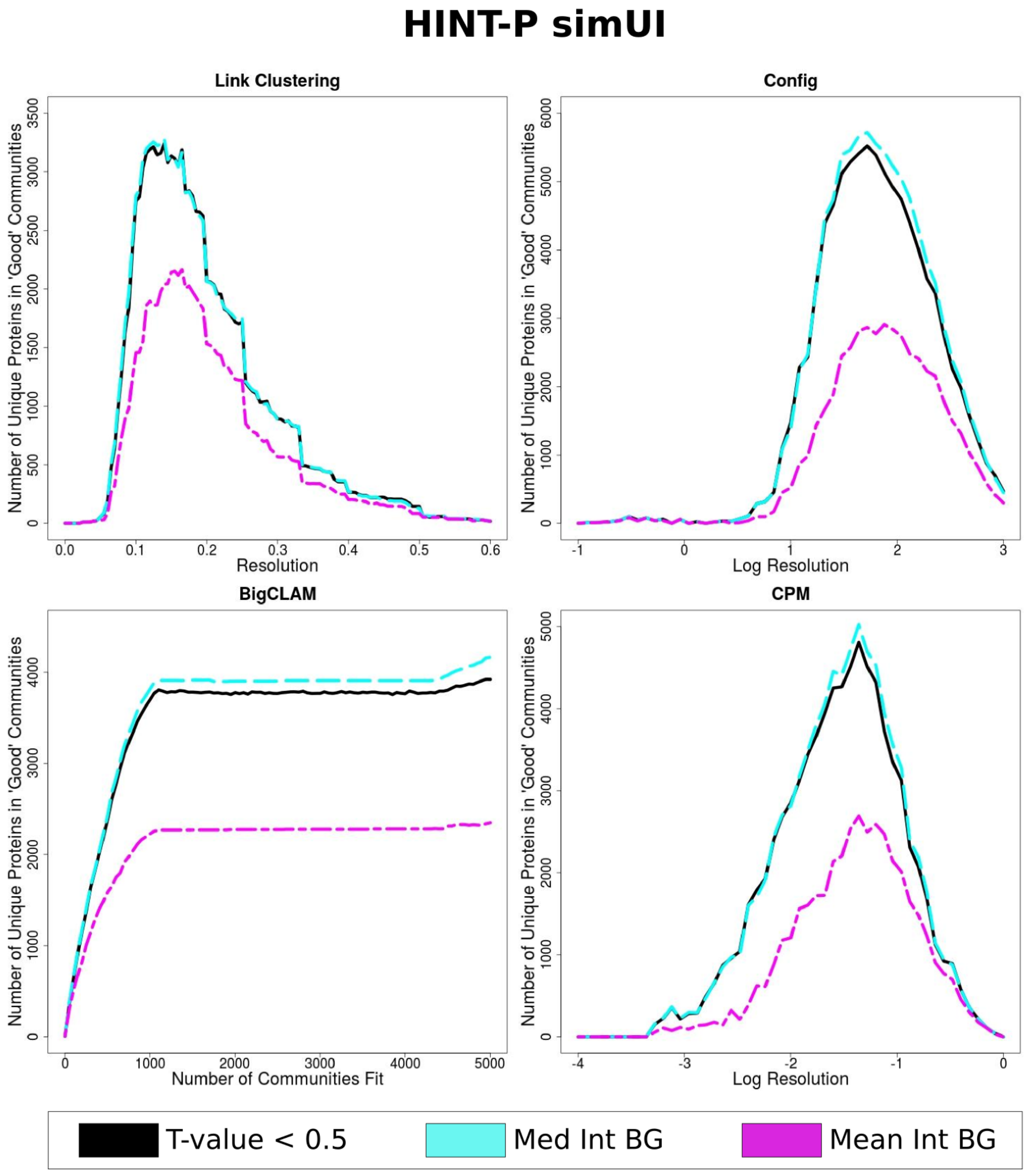

Fig S.10: Comparison of the number of unique proteins in significant communities of size 6 - 35 by T-value and functional homogeneity. The data was generated as in Figures S.4 and S.5 using the simUI semantic similarity measure on HINT-P. Using simUI, the number of unique proteins in T-value-significant communities is again consistently higher than in functional-homogeneity-significant communities for the qualitatively similar thresholds indicated by the black and cyan lines.

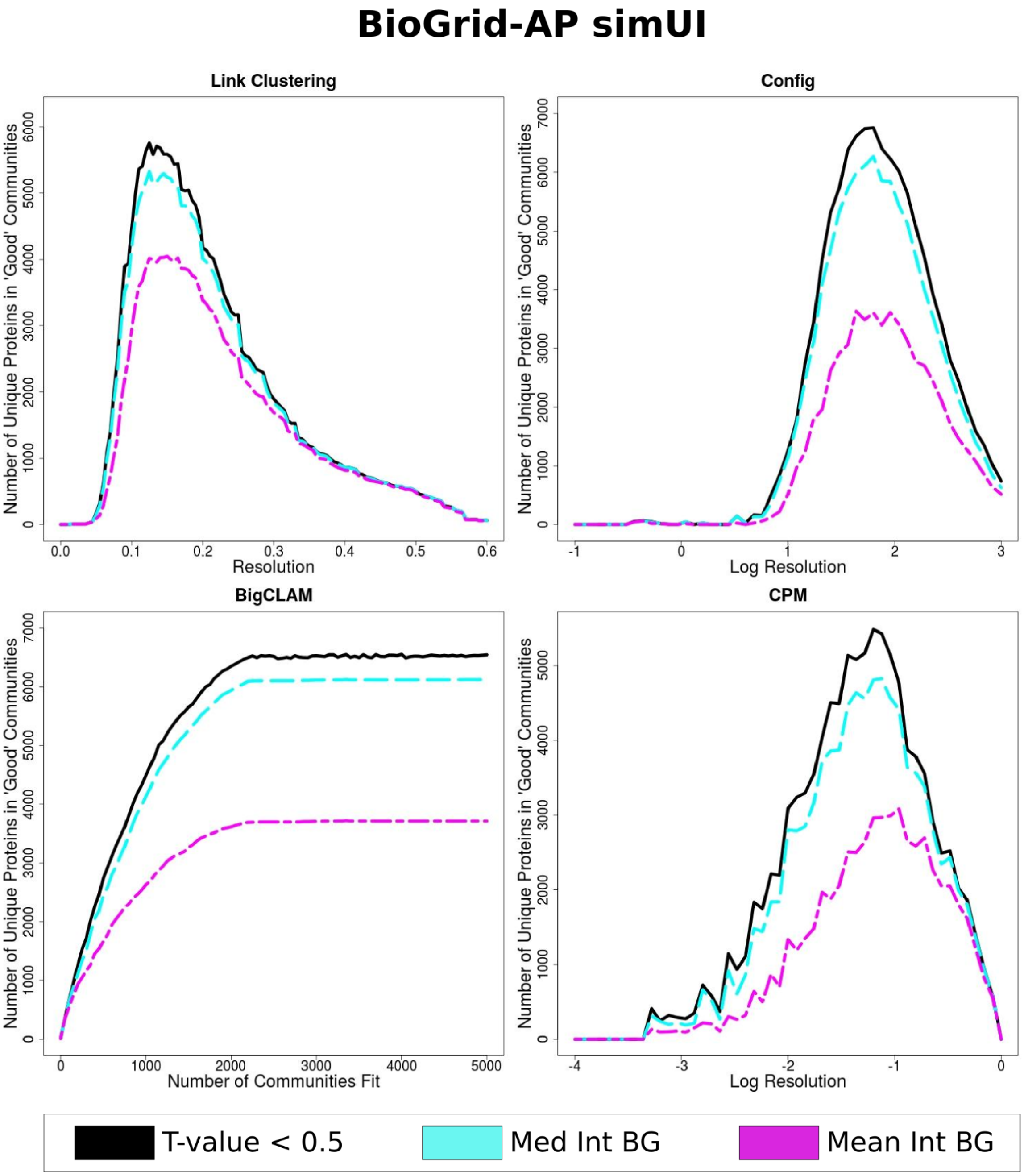

Fig S.11: Comparison of the number of unique proteins in significant communities of size 6 - 35 by T-value and functional homogeneity. The data was generated as in Figures S.4 and S.5 using the simUI semantic similarity measure on BioGrid-AP. Using simUI, the number of unique proteins in T-value-significant communities is again consistently higher than in functional-homogeneity-significant communities for the qualitatively similar thresholds indicated by the black and cyan lines.

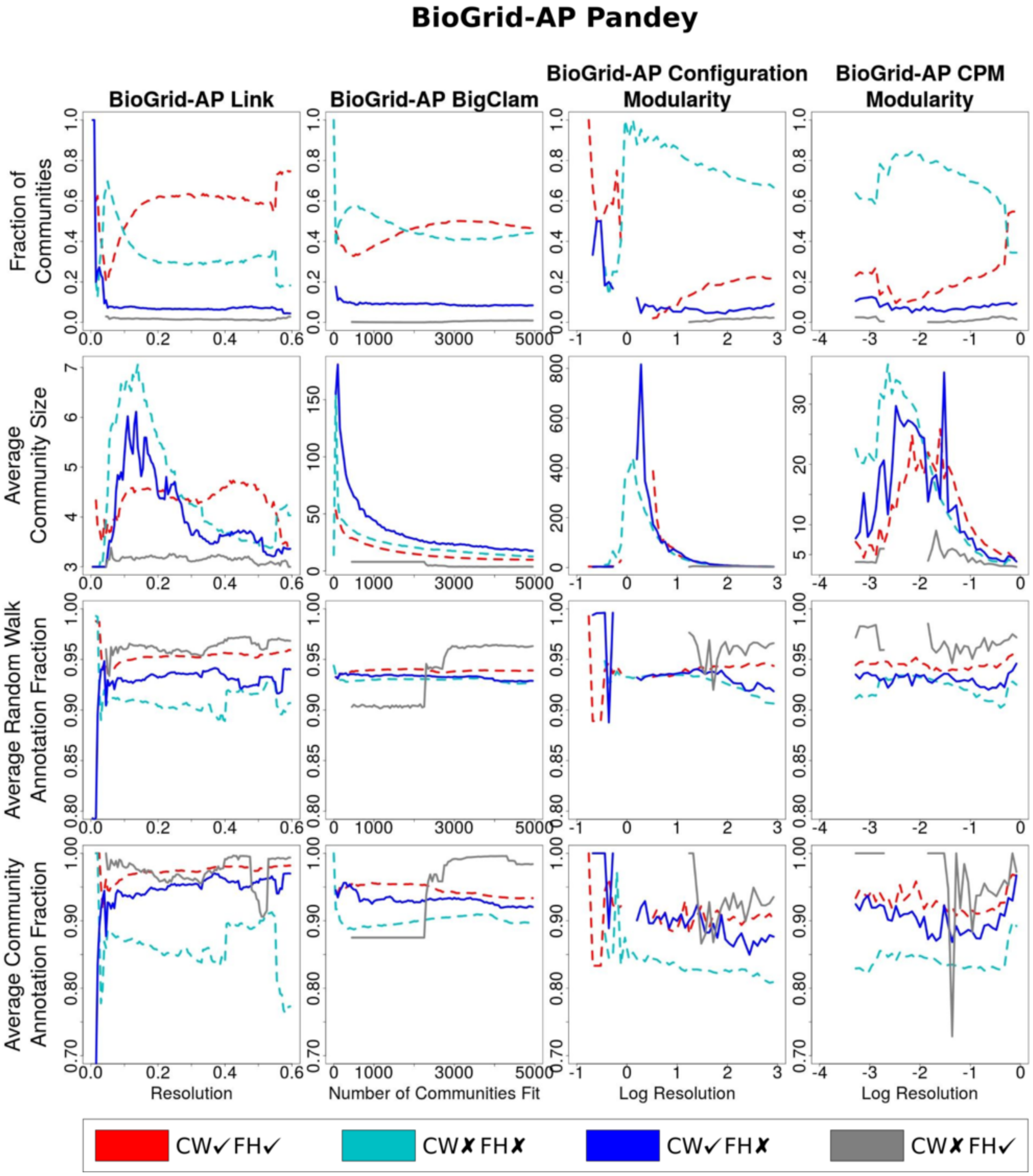

Fig S.12: Community statistics for BioGrid-AP communities generated by four multi-resolution community detection methods. Communities were divided into four groups depending on whether they were accepted or rejected by a T-value threshold of 0.5 and a functional homogeneity threshold of the median interaction background using the Pandey measure. Communities only accepted by T-value or functional homogeneity thresholds are plotted as blue and grey lines respectively, and communities accepted or rejected by both methods are shown as red or turquoise dotted lines respectively. The fraction of communities that fall into these categories is shown in the top row, with the following rows showing the average community size, the average level of annotation of the community, and the average annotation level of the communities' environments. The data in the lower two rows of graphs are only shown for comparison fraction ranges of 0.6 - 1.0 and 0.75 - 1.0 to emphasize the comparison between the data sets. As communities accepted only by functional homogeneity (grey lines) are the smallest set, summary statistics calculated for this set can be very variable. Taking these effects into account, the data suggests that functional homogeneity selects for smaller communities in well-annotated environments which also have a high level of annotation themselves.

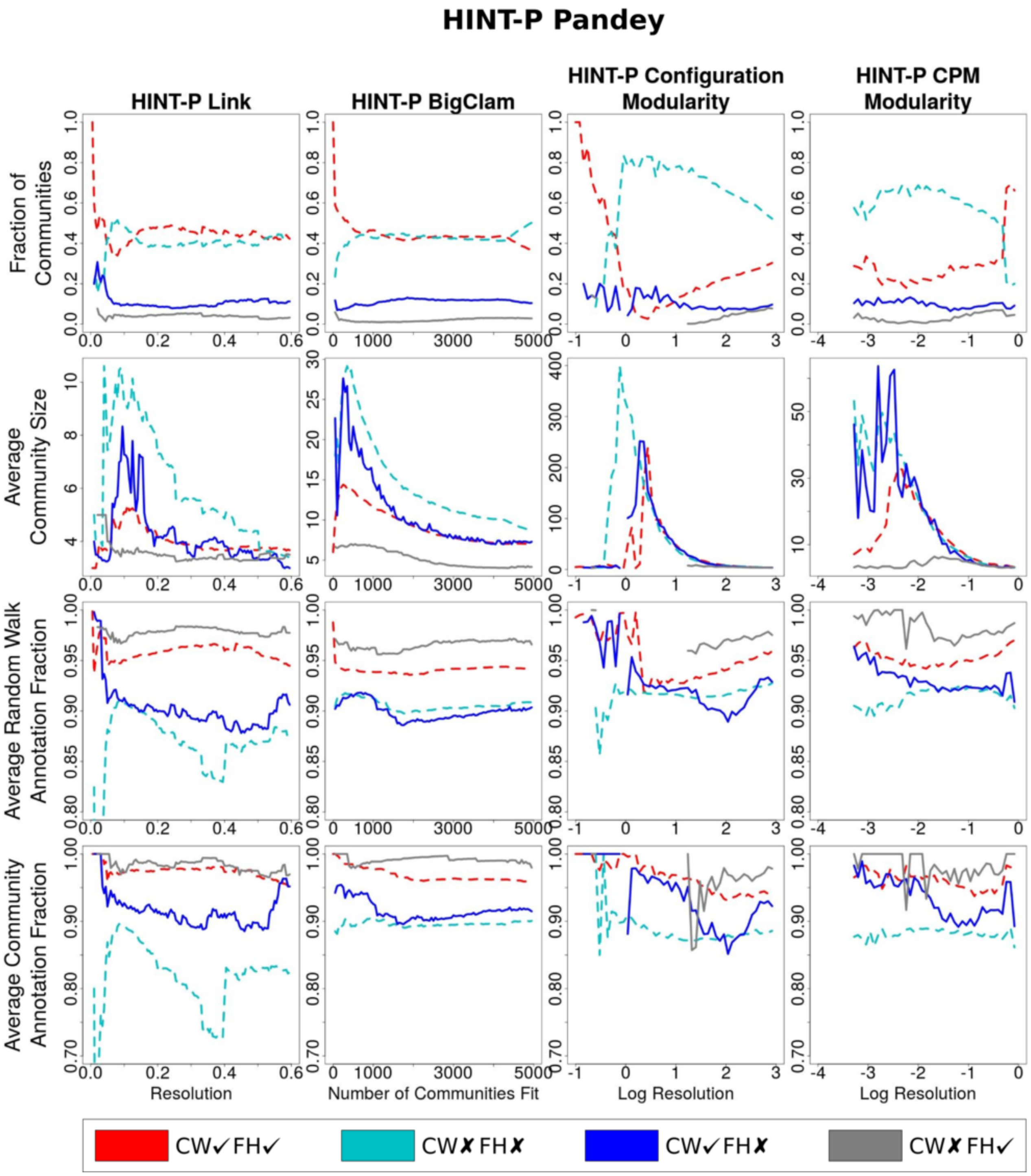

Fig S.13: Community statistics for HINT-P communities generated by four multi-resolution community detection methods. The data shown was generated as in Figure S.12 and suggests that functional homogeneity selects for smaller communities in well-annotated environments which also have a high level of annotation themselves. In contrast, T-value significant communities tend to have a broad distribution in the investigated statistics as seen by the lines representing CommWalker accepted communities (red dashed line and blue line). CommWalker can thus detect functionally significant communities even in ranges of the investigated network statistics in which communities tend to be rejected.

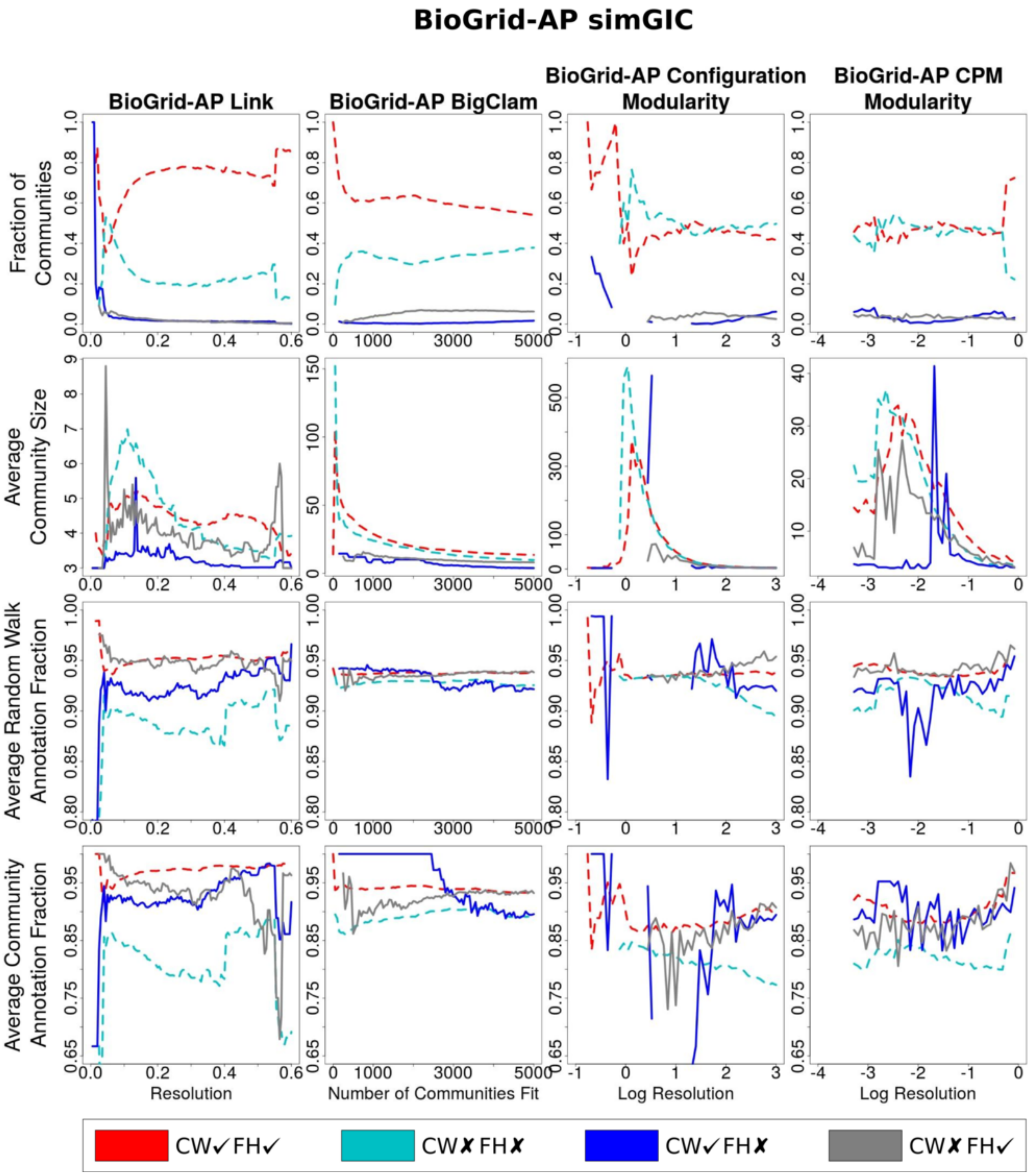

Fig S.14: Community statistics for BioGrid-AP communities generated by four multi-resolution community detection methods. The data shown was generated as in Figure S.12 using simGIC and suggests that functional homogeneity selects for smaller communities in well-annotated environments which also have a high level of annotation themselves. In contrast, T-value significant communities tend to have a broad distribution in the investigated statistics as seen by the lines representing CommWalker accepted communities (red dashed line and blue line). CommWalker can thus detect functionally significant communities even in ranges of the investigated network statistics in which communities tend to be rejected.

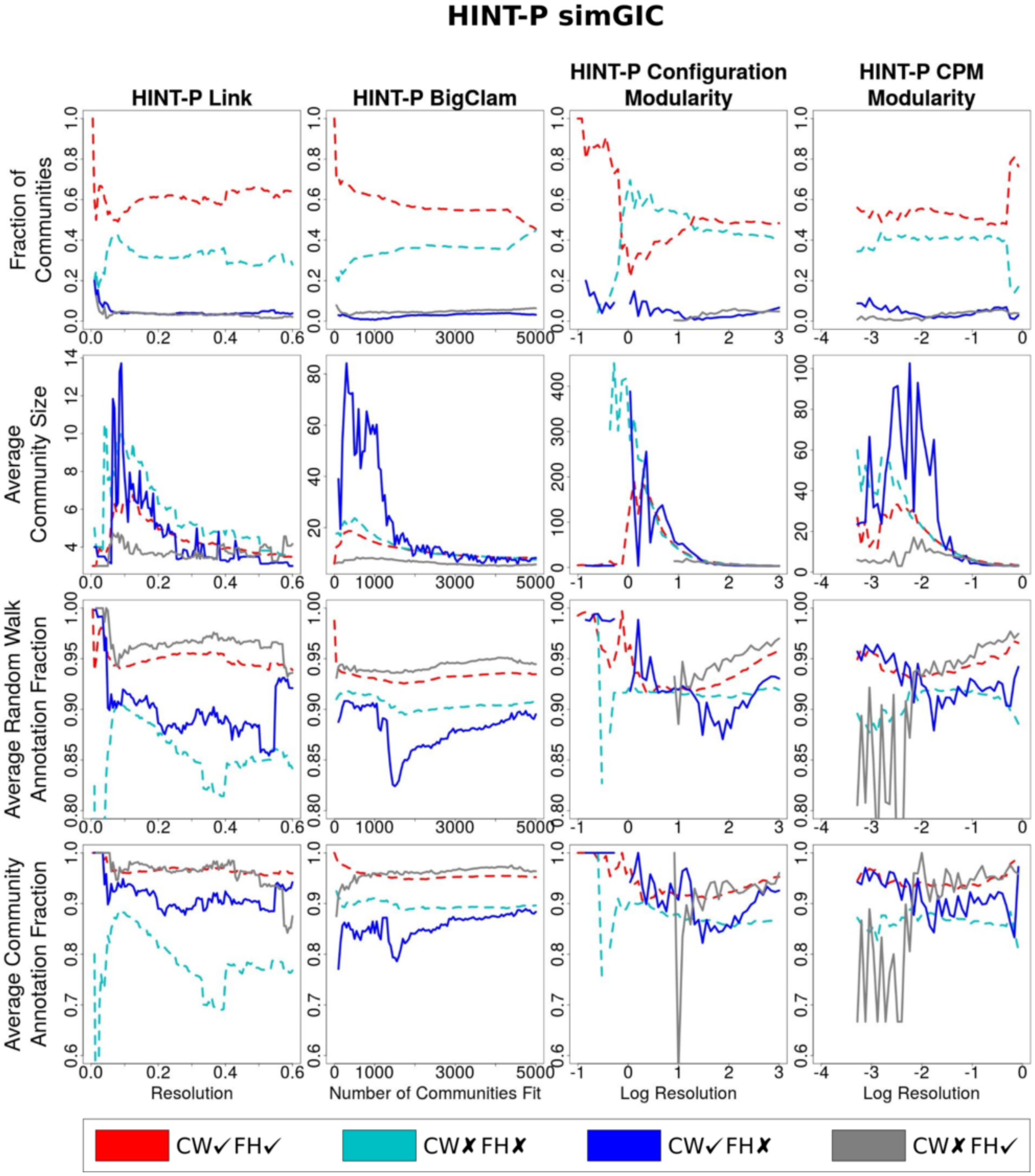

Fig S.15: Community statistics for HINT-P communities generated by four multi-resolution community detection methods. The data shown was generated as in Figure S.12 using simGIC and suggests that functional homogeneity selects for smaller communities in well-annotated environments which also have a high level of annotation themselves. In contrast, T-value significant communities tend to have a broad distribution in the investigated statistics as seen by the lines representing CommWalker accepted communities (red dashed line and blue line). CommWalker can thus detect functionally significant communities even in ranges of the investigated network statistics in which communities tend to be rejected.

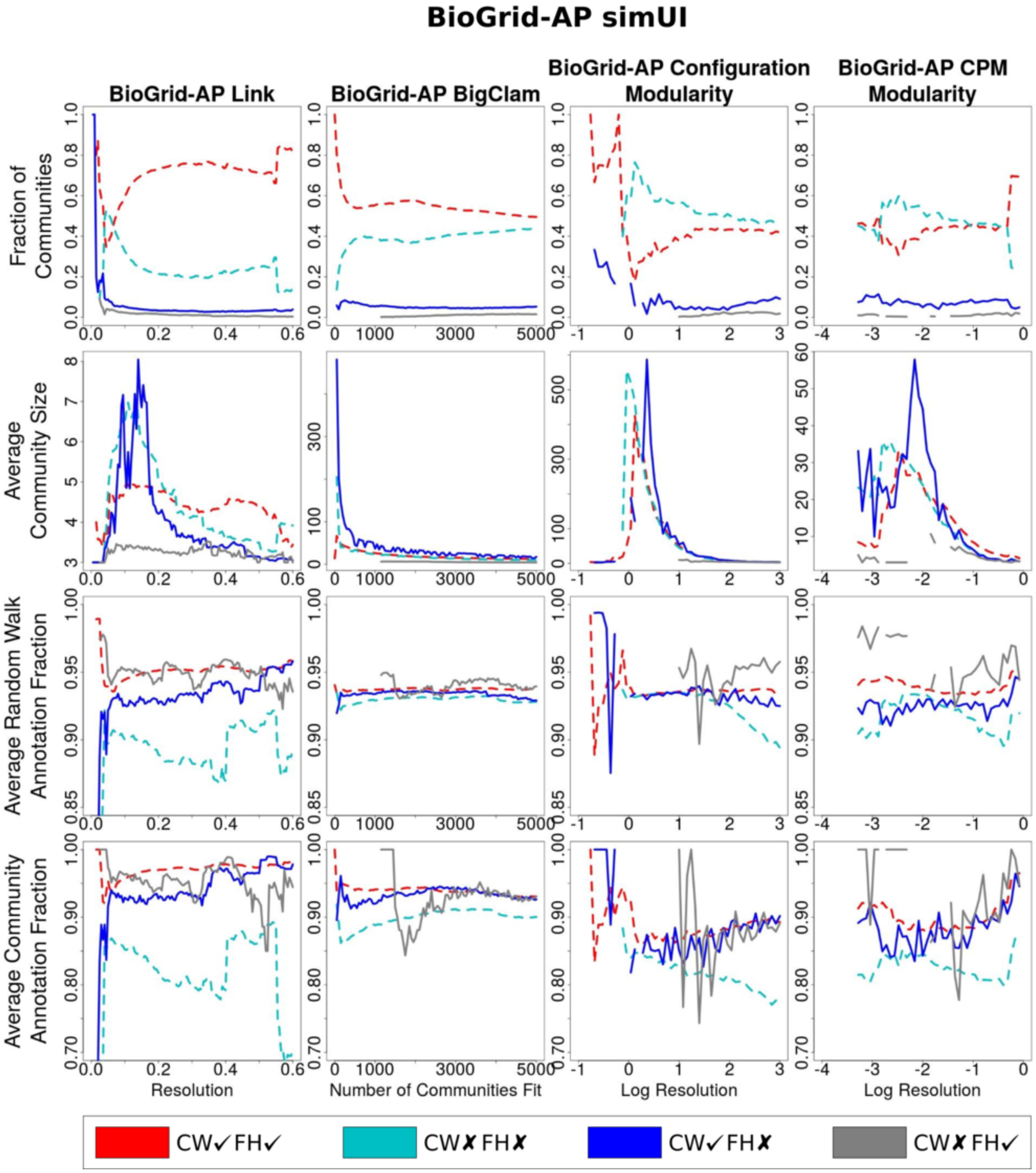

Fig S.16: Community statistics for BioGrid-AP communities generated by four multi-resolution community detection methods. The data shown was generated as in Figure S.12 using simUI and suggests that functional homogeneity selects for smaller communities in well-annotated environments which also have a high level of annotation themselves. In contrast, T-value significant communities tend to have a broad distribution in the investigated statistics as seen by the lines representing CommWalker accepted communities (red dashed line and blue line). CommWalker can thus detect functionally significant communities even in ranges of the investigated network statistics in which communities tend to be rejected.

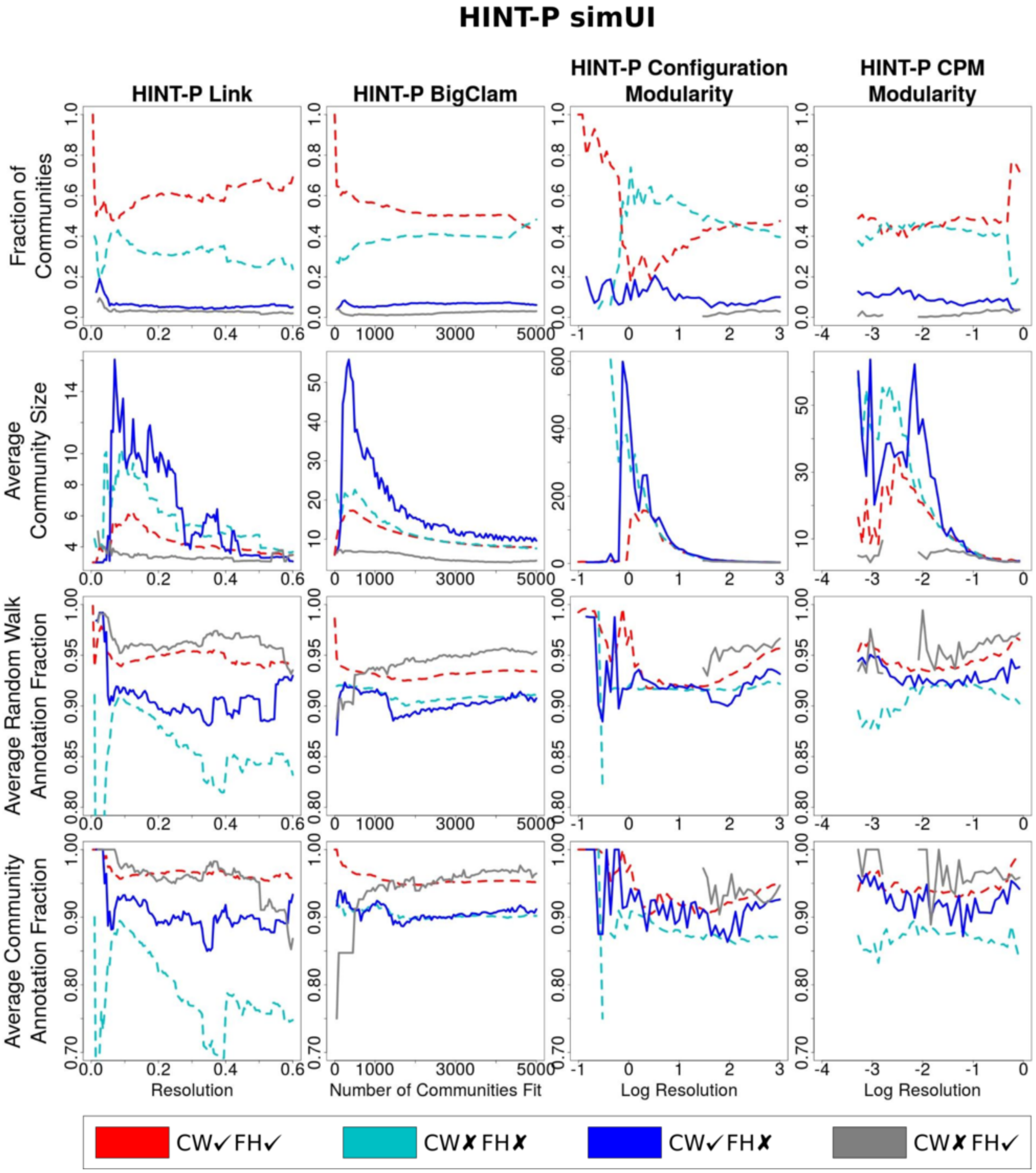

Fig S.17: Community statistics for HINT-P communities generated by four multi-resolution community detection methods. The data shown was generated as in Figure S.12 using simUI and suggests that functional homogeneity selects for smaller communities in well-annotated environments which also have a high level of annotation themselves. In contrast, T-value significant communities tend to have a broad distribution in the investigated statistics as seen by the lines representing CommWalker accepted communities (red dashed line and blue line). CommWalker can thus detect functionally significant communities even in ranges of the investigated network statistics in which communities tend to be rejected.

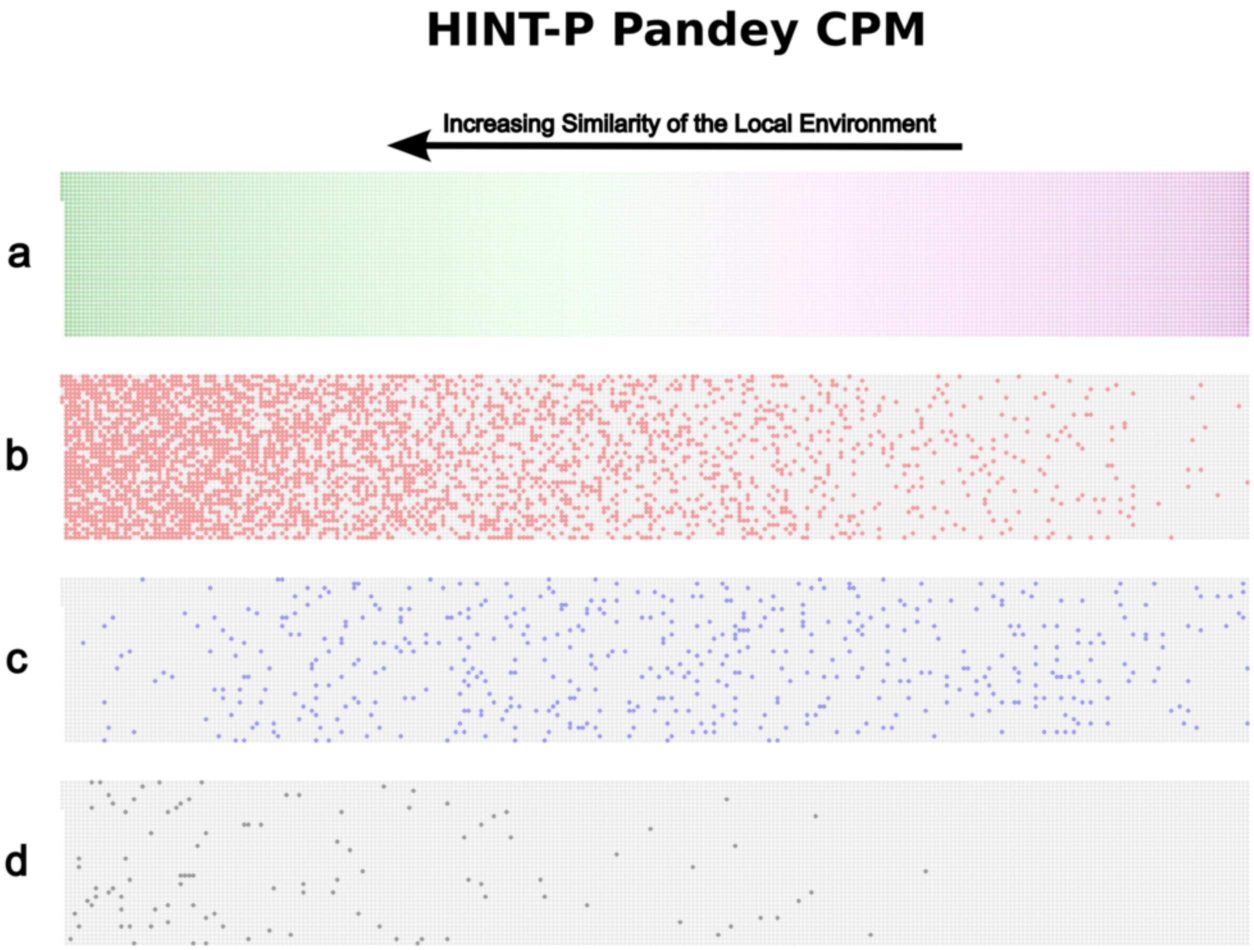

Fig S.18: Comparison of the environments of proteins in communities identified as biologically relevant by CommWalker and functional homogeneity. Nodes in HINT-P were ordered by their Pandey semantic similarity with nodes in their vicinity as shown in (a). Communities were generated by Constant Potts model Modularity Maximization at the resolution where the maximum number of proteins are found in functionally significant communities by a T-value threshold of 0.5 (log resolution = −1.36, cf. Figure S.4). On this network layout the proteins in communities identified as functionally significant by CommWalker and functional homogeneity in red (b), by only CommWalker in blue (c), and by only functional homogeneity in black (d) are shown. The further left the coloured nodes are, the higher the semantic similarity of their environment.

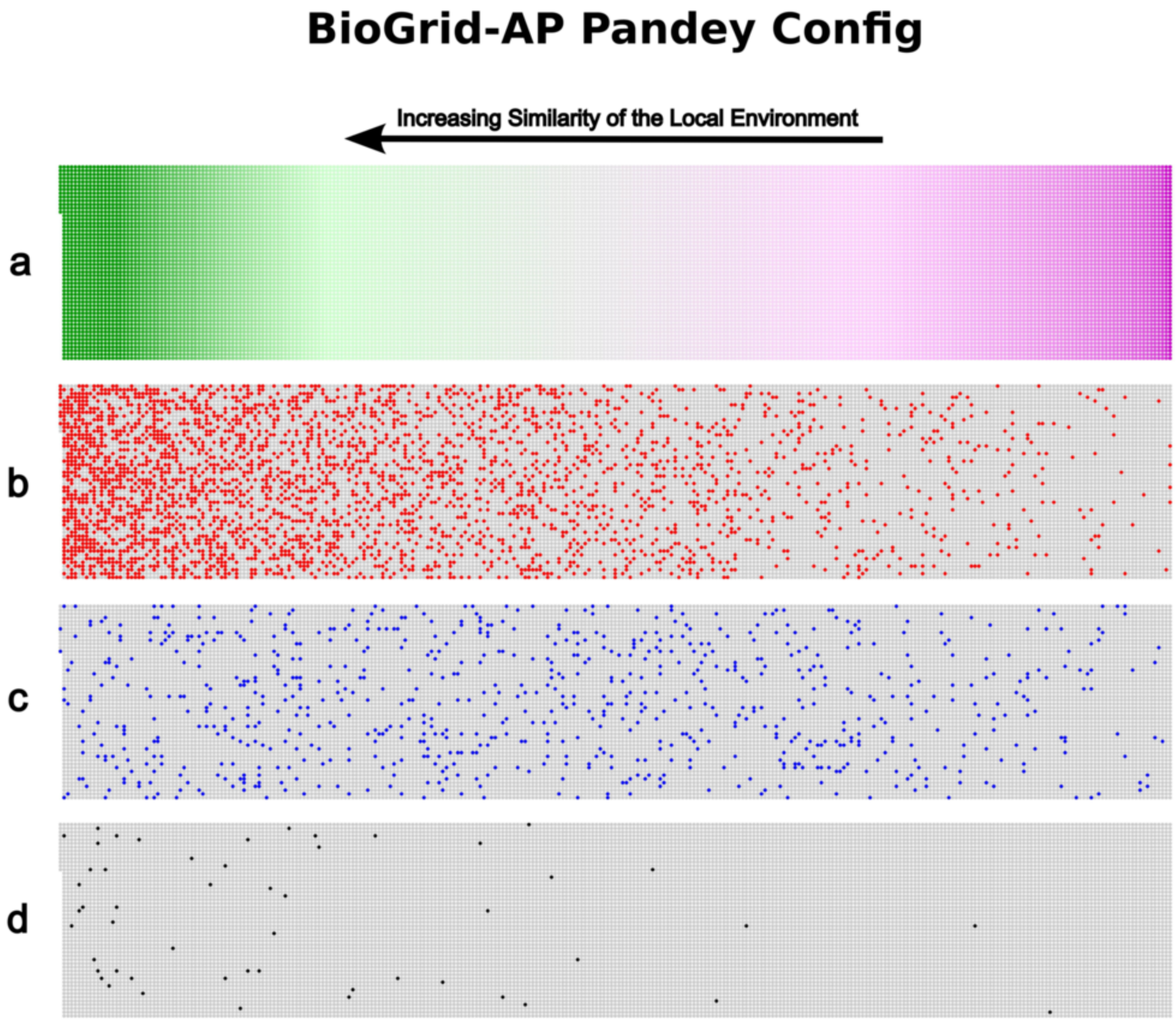

Fig S.19: Comparison of the environments of proteins in communities identified as biologically relevant by CommWalker and functional homogeneity. The data shown was generated as in Figure S.18 using configuration model Modularity Maximization on BioGrid-AP with the Pandey measure. Proteins are ordered by their semantic similairty with nodes in their vicinity (a). On this network layout, the proteins in communities identified as functionally significant by CommWalker and functional homogeneity in red (b), by only CommWalker in blue (c), and by only functional homogeneity in black (d) are shown. The further left the coloured nodes are, the higher the semantic similarity of their environment.

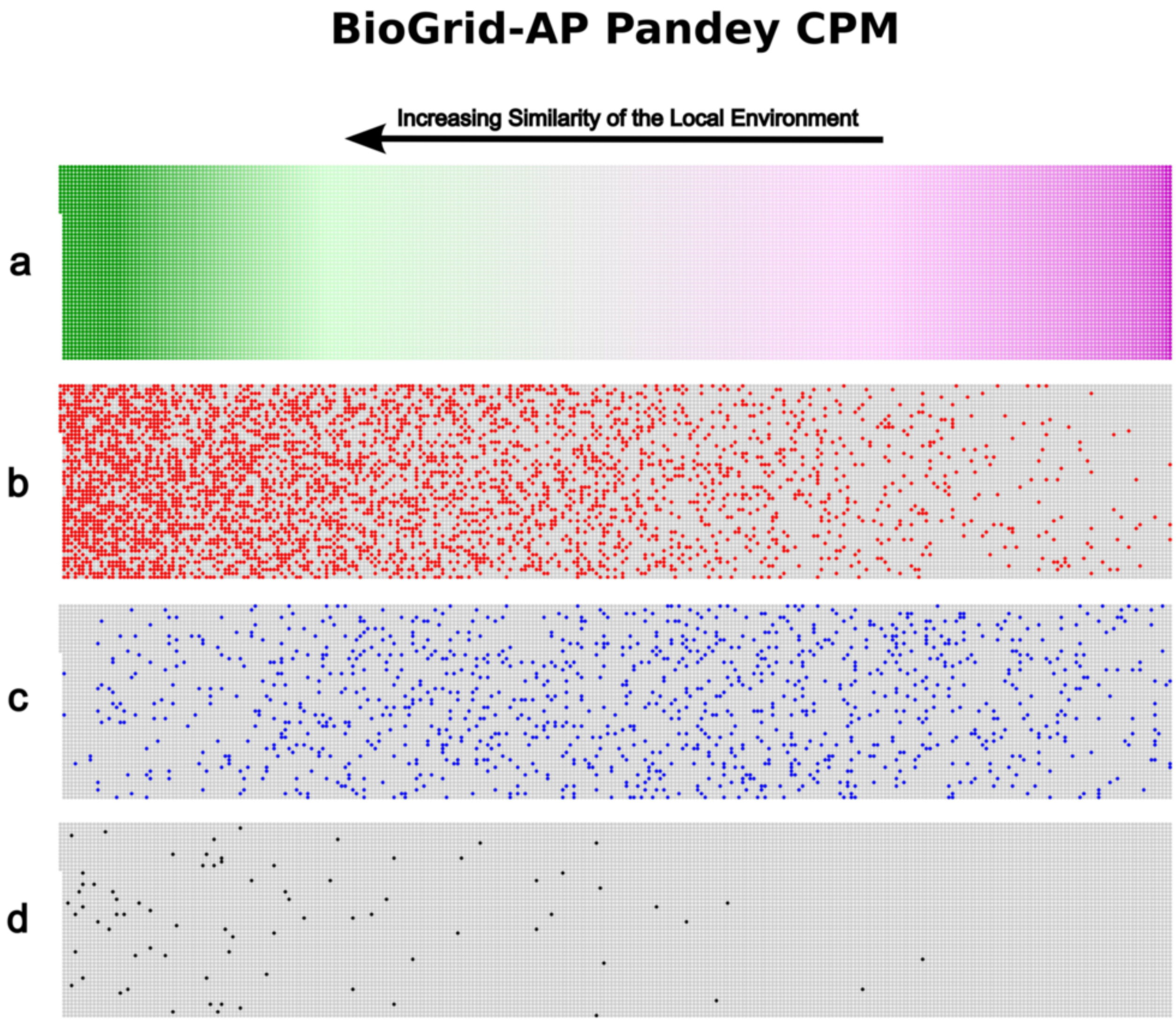

Fig S.20: Comparison of the environments of proteins in communities identified as biologically relevant by CommWalker and functional homogeneity. The data shown was generated as in Figure S.18 using Constant Potts model Modularity Maximization on BioGrid-AP with the Pandey measure. Proteins are ordered by their semantic similairty with nodes in their vicinity (a). On this network layout, the proteins in communities identified as functionally significant by CommWalker and functional homogeneity in red (b), by only CommWalker in blue (c), and by only functional homogeneity in black (d) are shown. The further left the coloured nodes are, the higher the semantic similarity of their environment.

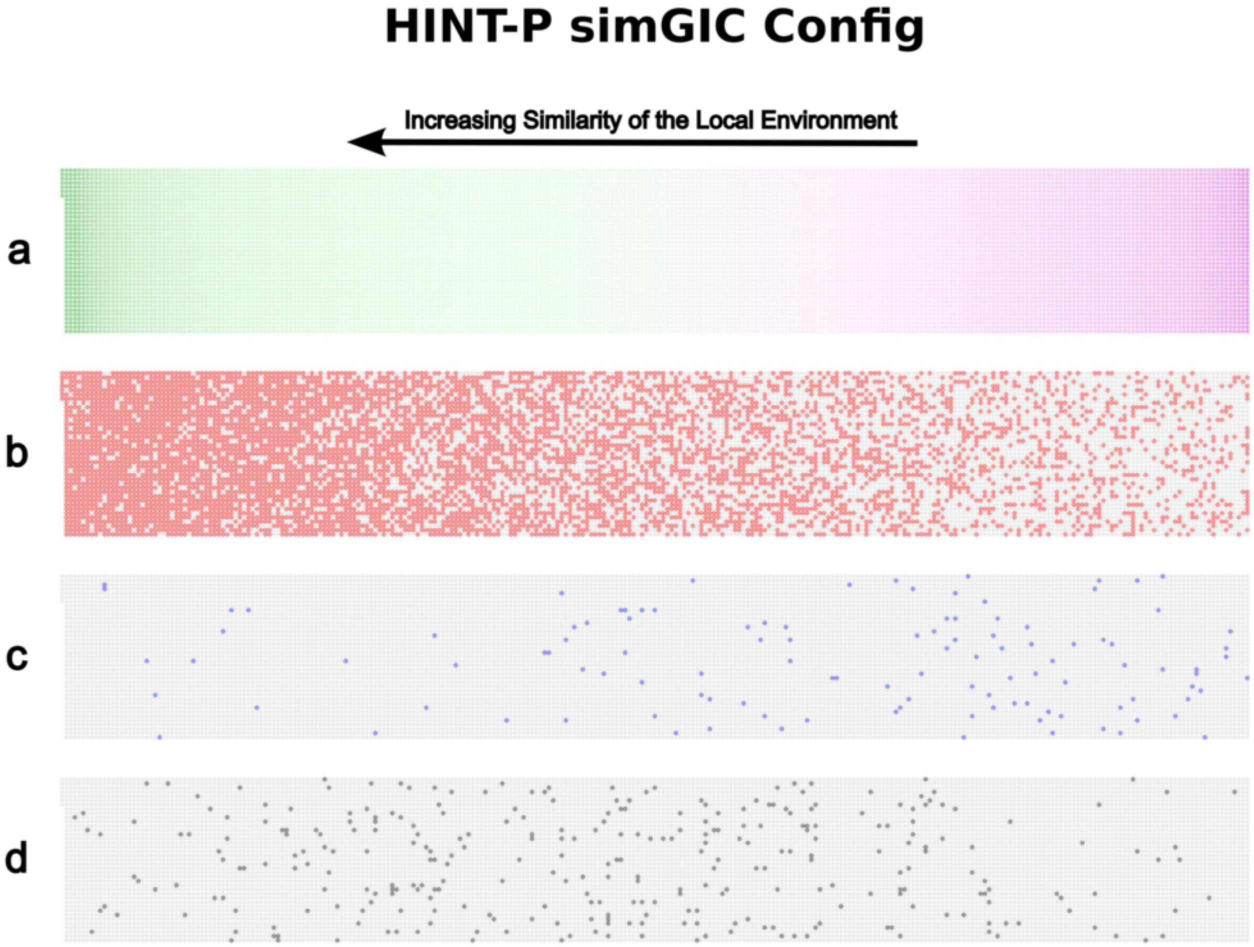

Fig S.21: Comparison of the environments of proteins in communities identified as biologically relevant by CommWalker and functional homogeneity. The data shown was generated as in Figure S.18 using configuration model Modularity Maximization on HINT-P with the simGIC semantic similarity measure. Proteins are ordered by their semantic similairty with nodes in their vicinity (a). On this network layout, the proteins in communities identified as functionally significant by CommWalker and functional homogeneity in red (b), by only CommWalker in blue (c), and by only functional homogeneity in black (d) are shown. The further left the coloured nodes are, the higher the semantic similarity of their environment.

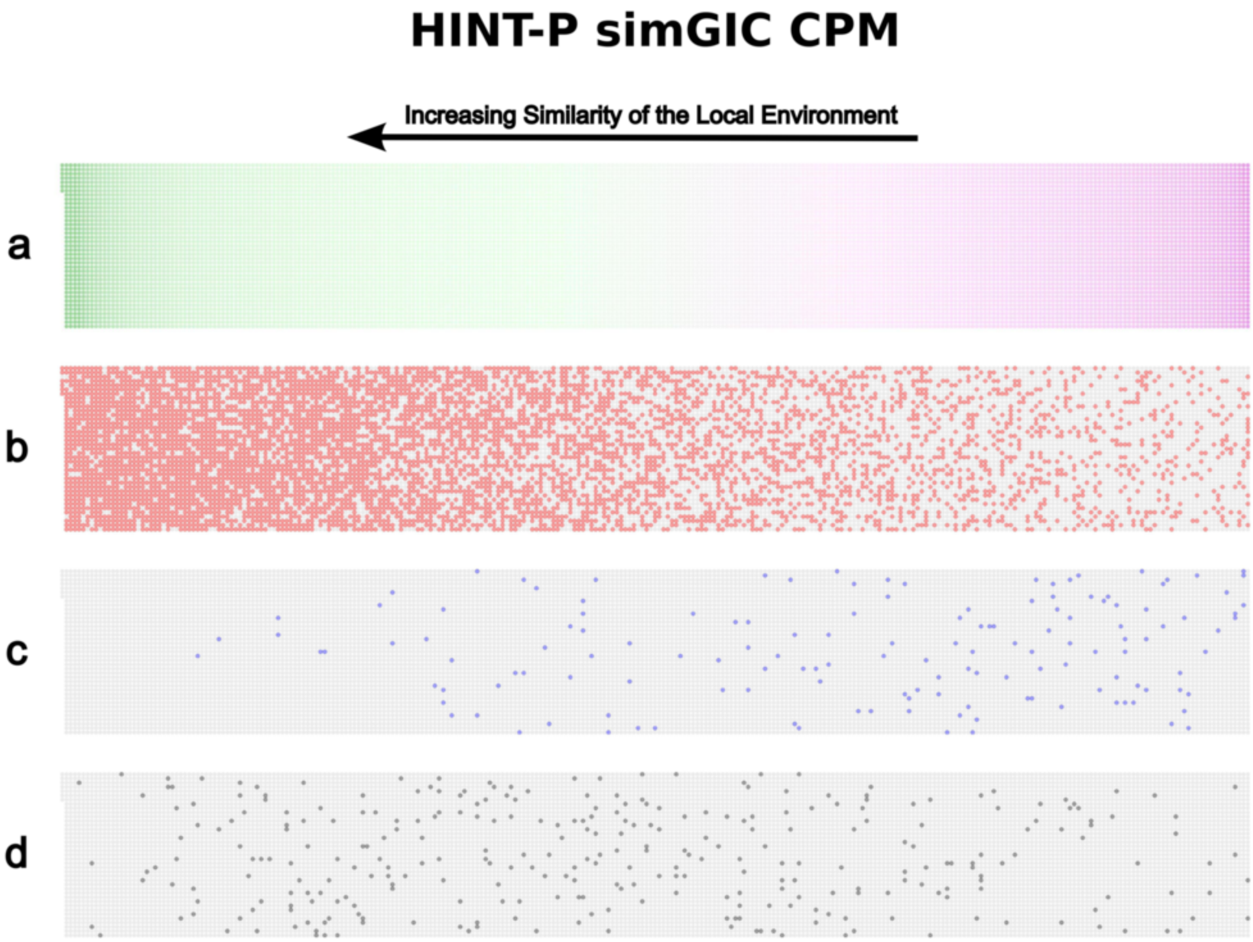

Fig S.22: Comparison of the environments of proteins in communities identified as biologically relevant by CommWalker and functional homogeneity. The data shown was generated as in Figure S.18 using Constant Potts model Modularity Maximization on HINT-P with the simGIC semantic similarity measure. Proteins are ordered by their semantic similairty with nodes in their vicinity (a). On this network layout, the proteins in communities identified as functionally significant by CommWalker and functional homogeneity in red (b), by only CommWalker in blue (c), and by only functional homogeneity in black (d) are shown. The further left the coloured nodes are, the higher the semantic similarity of their environment.

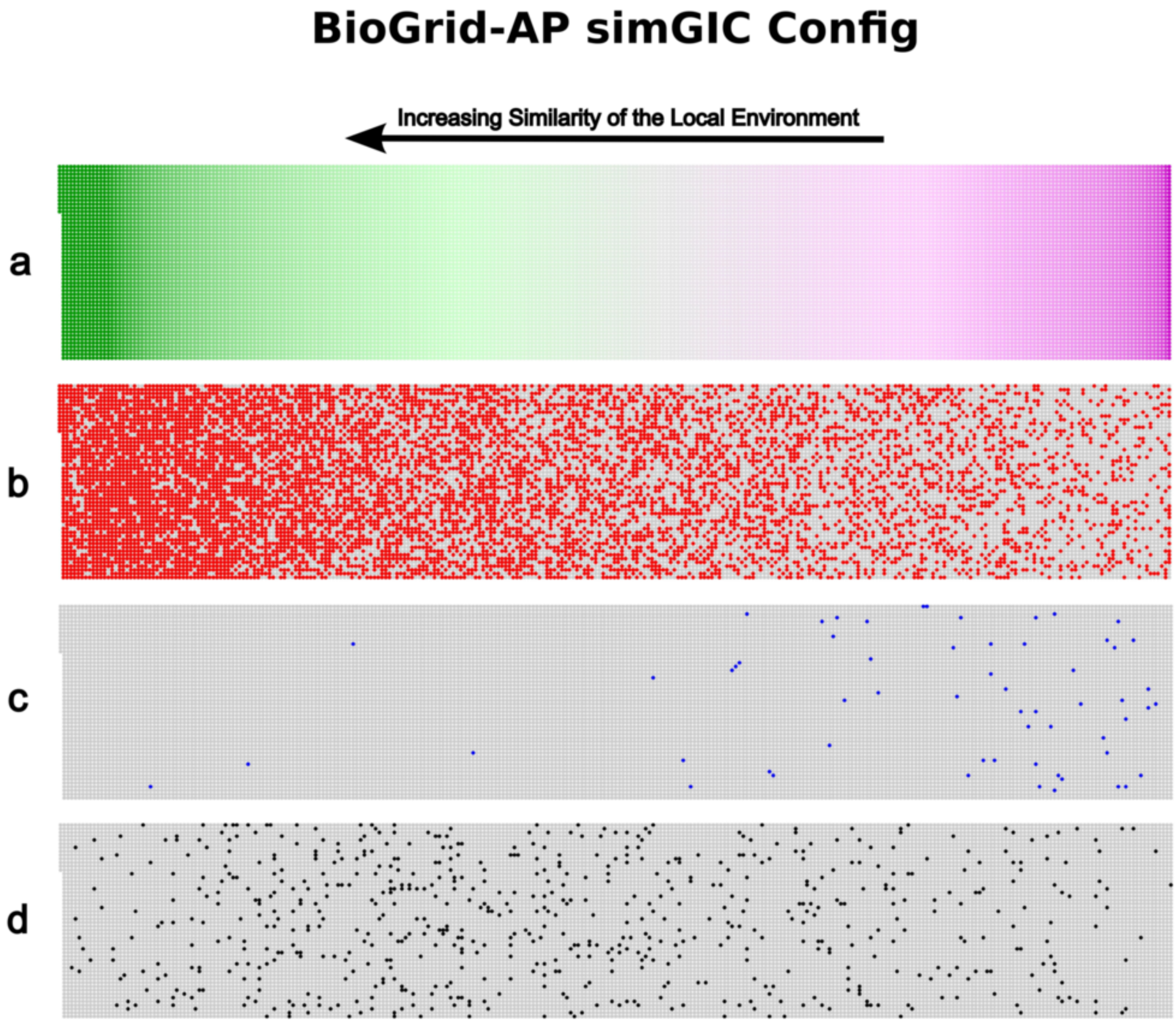

Fig S.23: Comparison of the environments of proteins in communities identified as biologically relevant by CommWalker and functional homogeneity. The data shown was generated as in Figure S.18 using configuration model Modularity Maximization on BioGrid-AP with the simGIC semantic similarity measure. Proteins are ordered by their semantic similairty with nodes in their vicinity (a). On this network layout, the proteins in communities identified as functionally significant by CommWalker and functional homogeneity in red (b), by only CommWalker in blue (c), and by only functional homogeneity in black (d) are shown. The further left the coloured nodes are, the higher the semantic similarity of their environment.

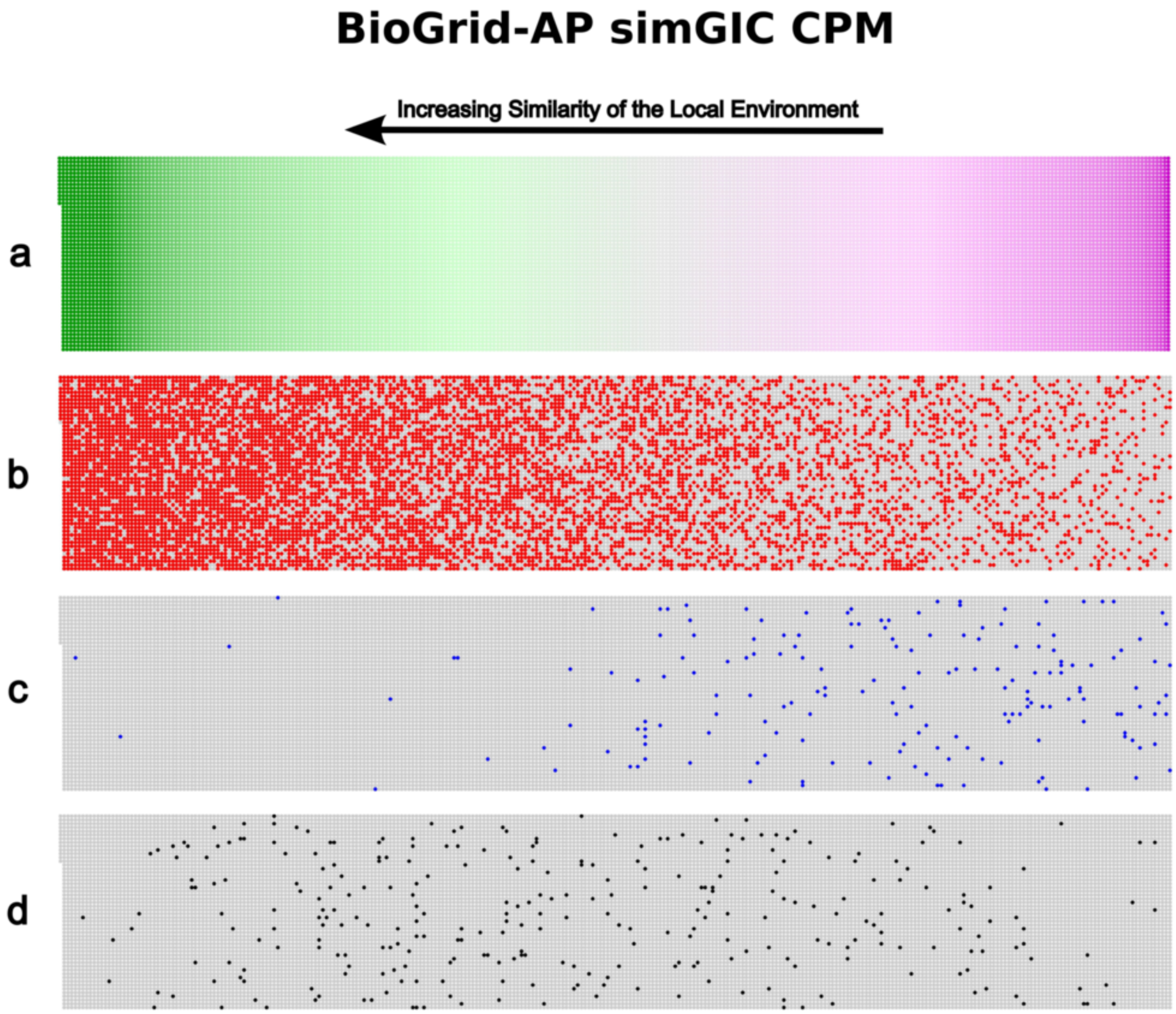

Fig S.24: Comparison of the environments of proteins in communities identified as biologically relevant by CommWalker and functional homogeneity. The data shown was generated as in Figure S.18 using Constant Potts model Modularity Maximization on BioGrid-AP with the simGIC semantic similarity measure. Proteins are ordered by their semantic similairty with nodes in their vicinity (a). On this network layout, the proteins in communities identified as functionally significant by CommWalker and functional homogeneity in red (b), by only CommWalker in blue (c), and by only functional homogeneity in black (d) are shown. The further left the coloured nodes are, the higher the semantic similarity of their environment.

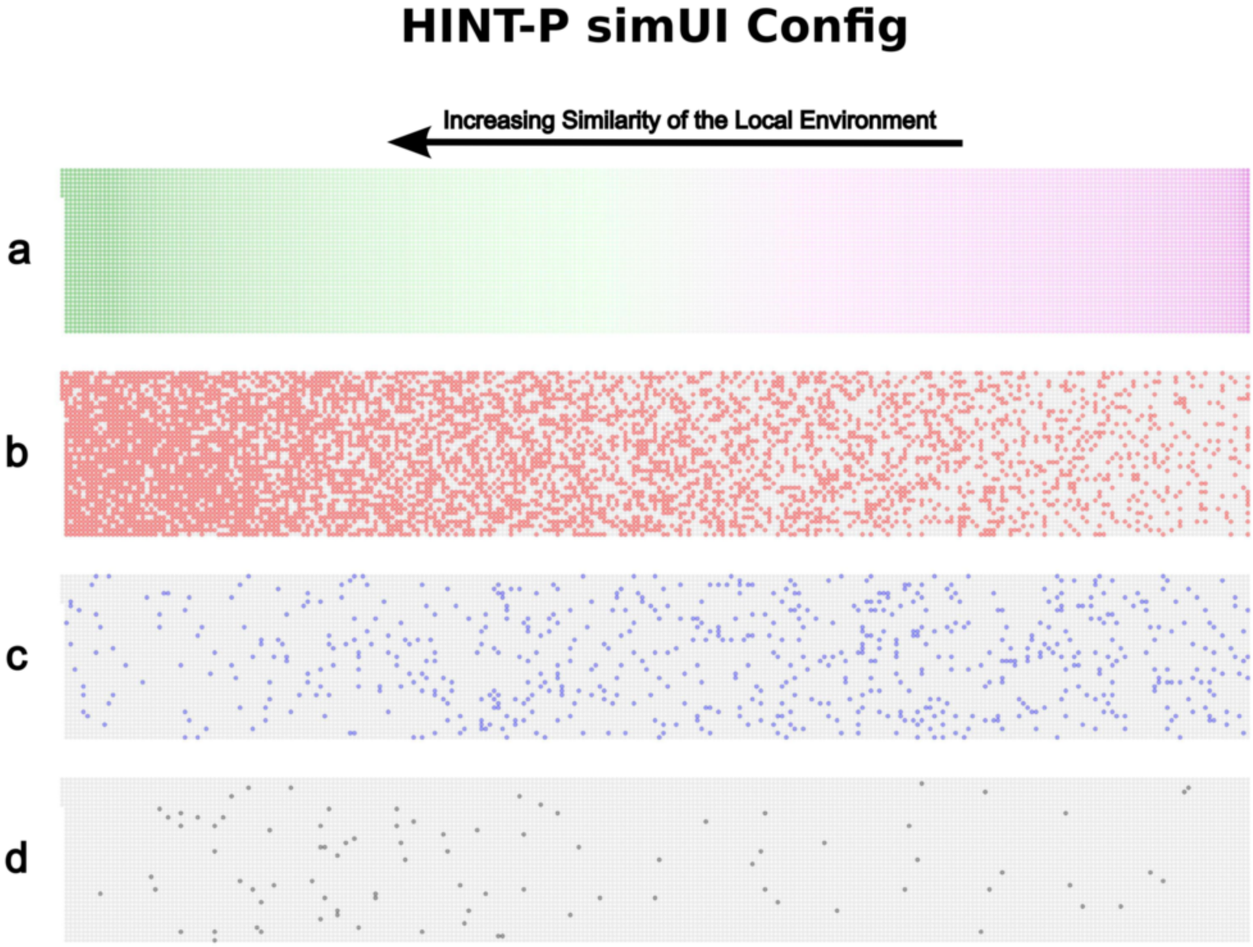

Fig S.25: Comparison of the environments of proteins in communities identified as biologically relevant by CommWalker and functional homogeneity. The data shown was generated as in Figure S.18 using configuration model Modularity Maximization on HINT-P with the simUI semantic similarity measure. Proteins are ordered by their semantic similairty with nodes in their vicinity (a). On this network layout, the proteins in communities identified as functionally significant by CommWalker and functional homogeneity in red (b), by only CommWalker in blue (c), and by only functional homogeneity in black (d) are shown. The further left the coloured nodes are, the higher the semantic similarity of their environment.

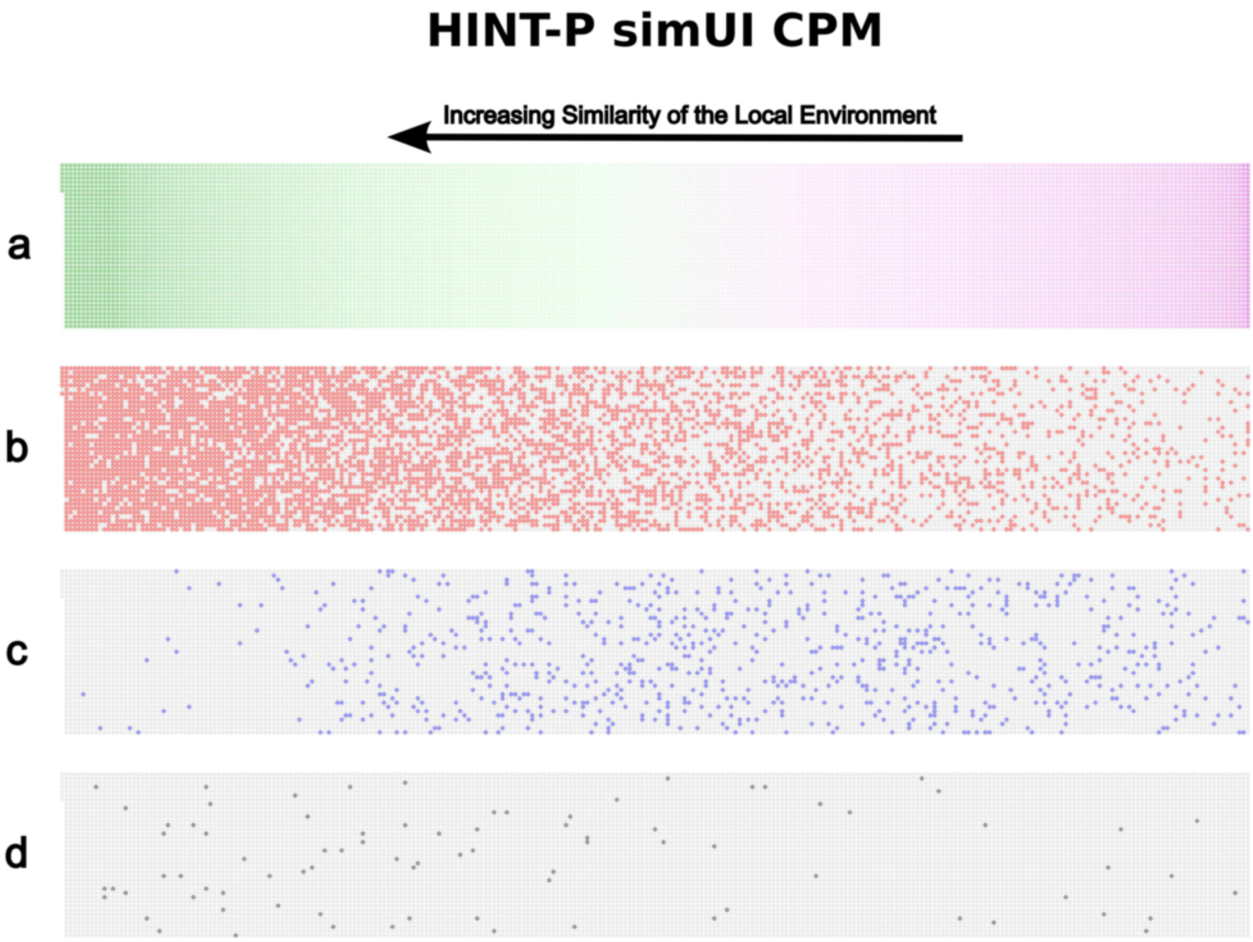

Fig S.26: Comparison of the environments of proteins in communities identified as biologically relevant by CommWalker and functional homogeneity. The data shown was generated as in Figure S.18 using Constant Potts model Modularity Maximization on HINT-P with the simUI semantic similarity measure. Proteins are ordered by their semantic similairty with nodes in their vicinity (a). On this network layout, the proteins in communities identified as functionally significant by CommWalker and functional homogeneity in red (b), by only CommWalker in blue (c), and by only functional homogeneity in black (d) are shown. The further left the coloured nodes are, the higher the semantic similarity of their environment.

5 Appendix E: Module Validation Results

We validated the modules accepted by CommWalker by the level of co-expression of genes grouped together. Co-expression of two genes was quantified by the absolute value of Pearson's correlation coefficient of the expression levels of these genes across samples (cf. *Methods*). The co-expression level of a community was computed by averaging the pairwise co-expression scores of the proteins in this community. As the relationship between co-expression and functional relatedness is complex (Lee *et al.*, 2004; Li and Biggin, 2015; van Noort *et al.*, 2003; Zhou *et al.*, 2002), we do not expect all data sets to have optimally captured gene co-expression. Thus, it is necessary to choose the data set that best selects for co-expressed genes for our validation.

We performed an evaluation of the data sets by comparing the co-expression of the community sets accepted by CommWalker and/or functional homogeneity, to the co-expression scores of random walks on the respective PINs. 1000 random walks of length 6 were performed from each node in the respective networks to generate a background co-expression score distribution of random proxy communities. To assess how similar the random walk co-expression is to the community set co-expression, we computed the fraction of the random walk co-expression scores that exceeded a threshold ("overlap score"). This threshold was set at the 25% quantile of the community set co-expression score distribution. The results of this investigation are shown in Table S.3.

The computed overlap scores show that BioGrid-AP partitioned by link clustering using the Pandey measure to evaluate the resulting communities is the only data set with an overlap score below 15% for modules accepted by both methods. This data set thus best captures gene co-expression and was chosen for module validation. We repeated the analysis using the 10% quantile as threshold to calculate the overlap score (Table S.4), and drew the same conclusion.

We analysed the distribution of community set co-expression scores for this data set in Figure 4 in the paper, showing that the median co-expression level is substantially higher for those communities only accepted by CommWalker, than communities only accepted by functional homogeneity. To assess whether this observation was due to different community sizes in the two community sets, we also plotted the mean co-expression score of the community sets at different community sizes (Figure S.29). Figure S.29 shows that the observation holds true for most community sizes, apart from size 6, where there are only 90 data points. It should be noted that only the first two data points for the only functional homogeneity accepted data set contain over 1000 data points.

To further validate modules accepted by CommWalker we chose the largest module in this data set that was strongly rejected by functional homogeneity (FH < 5, instead of median semantic similarity of interacting proteins at 6.10552), but still clearly accepted by CommWalker (T-value < 0.25) and analysed it in more detail in the paper. Similarly, we also investigated the largest module that was strongly rejected by CommWalker (T-value > 0.6), while being clearly accepted by functional homogeneity (FH > 6.5). This module is a star community centered around STAT3, further containing the genes LEP, CEP120, NFKBIZ, HES5, and IL22RA1. Most of these genes were found to be involved with signalling and/or regulating transciption, which explains their interaction with STAT3. However, we found no closer connection between the low degree genes clustered toghether. The average number of GO BP annotations associated with the genes in this module is 257.17, which is significantly larger than the mean of 89.85 in BioGrid-AP. While this investigation cannot show that modules only accepted by functional homogeneity should not be considered, it does allow us to contrast the TRAPP module result discussed in the paper.

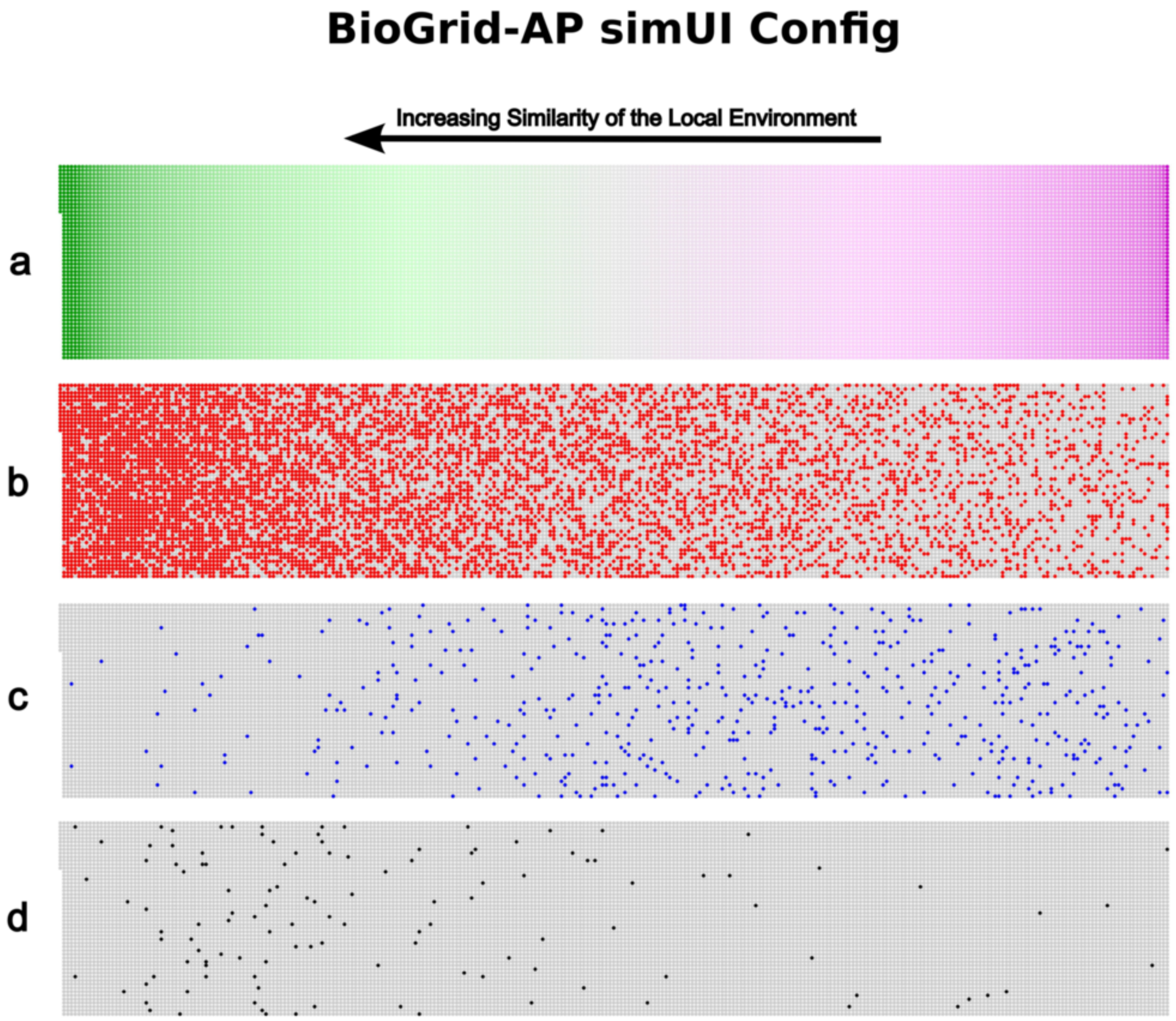

Fig S.27: Comparison of the environments of proteins in communities identified as biologically relevant by CommWalker and functional homogeneity. The data shown was generated as in Figure S.18 using configuration model Modularity Maximization on BioGrid-AP with the simUI semantic similarity measure. Proteins are ordered by their semantic similairty with nodes in their vicinity (a). On this network layout, the proteins in communities identified as functionally significant by CommWalker and functional homogeneity in red (b), by only CommWalker in blue (c), and by only functional homogeneity in black (d) are shown. The further left the coloured nodes are, the higher the semantic similarity of their environment.

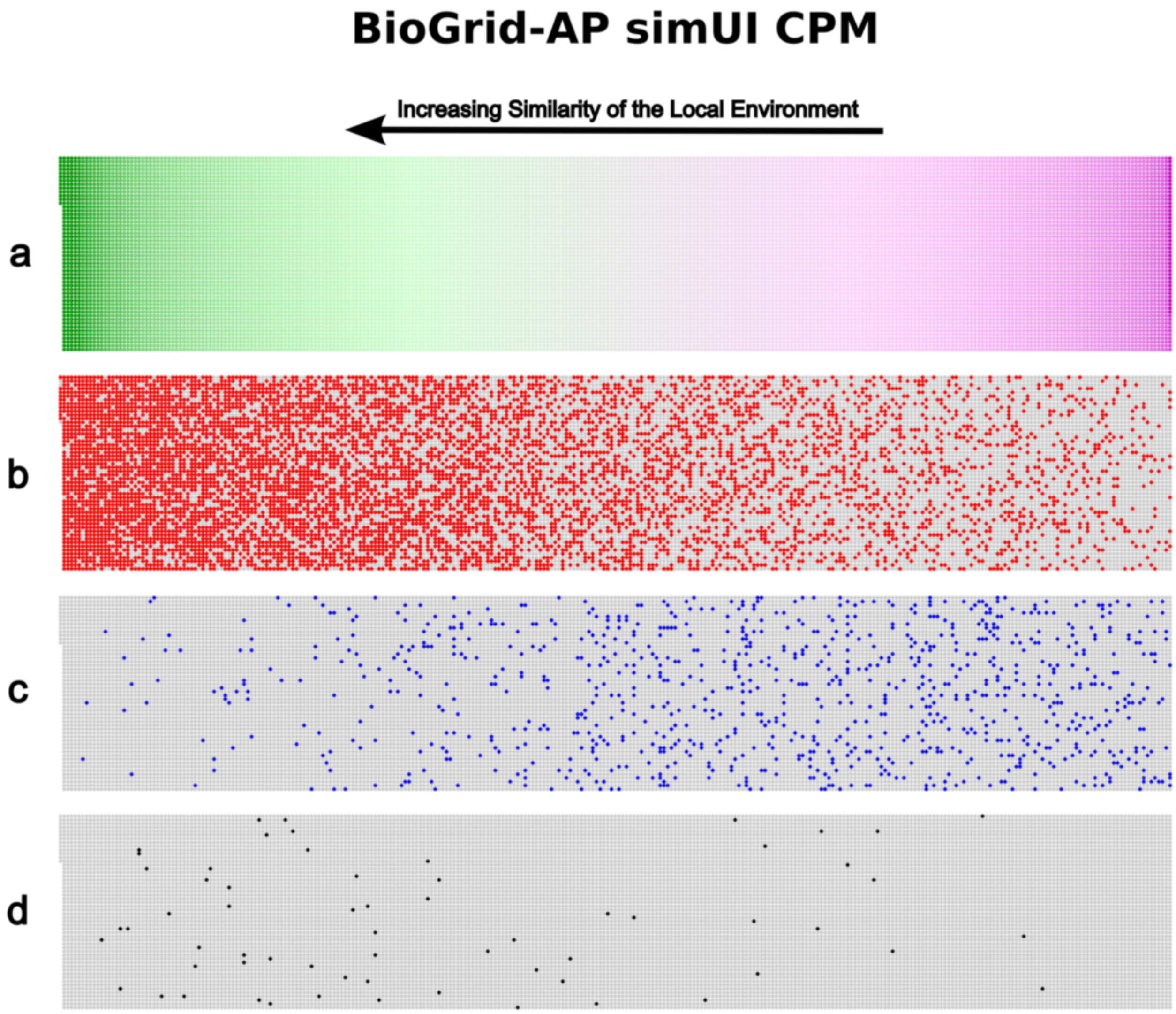

Fig S.28: Comparison of the environments of proteins in communities identified as biologically relevant by CommWalker and functional homogeneity. The data shown was generated as in Figure S.18 using Constant Potts model Modularity Maximization on BioGrid-AP with the simUI semantic similarity measure. Proteins are ordered by their semantic similairty with nodes in their vicinity (a). On this network layout, the proteins in communities identified as functionally significant by CommWalker and functional homogeneity in red (b), by only CommWalker in blue (c), and by only functional homogeneity in black (d) are shown. The further left the coloured nodes are, the higher the semantic similarity of their environment.

Table S.3: Overlap scores for data set selection for gene co-expression analysis.

Network	Comm Det	Sem Sim	Both acc	CW only	FH only	Both rej
HINT-P	Link	Pandey	0.243	0.264	0.441	0.316
		simUI	0.249	0.297	0.301	0.334
		simGIC	0.247	0.316	0.349	0.331
	BigClam	Pandey	0.304	0.276	0.451	0.302
		simUI	0.267	0.315	0.293	0.324
		simGIC	0.271	0.301	0.372	0.318
	Config	Pandey	0.332	0.334	0.361	0.319
		simUI	0.298	0.331	0.360	0.335
		simGIC	0.302	0.315	0.334	0.337
	CPM	Pandey	0.312	0.332	0.403	0.315
		simUI	0.292	0.333	0.339	0.335
		simGIC	0.300	0.369	0.349	0.334
BioGrid-AP	Link	Pandey	0.135	0.247	0.401	0.356
		simUI	0.151	0.366	0.361	0.397
		simGIC	0.160	0.395	0.361	0.401
	BigClam	Pandey	0.312	0.303	0.444	0.331
		simUI	0.287	0.338	0.273	0.351
		simGIC	0.291	0.414	0.309	0.360
	Config	Pandey	0.374	0.386	0.548	0.403
		simUI	0.351	0.410	0.384	0.432
		simGIC	0.356	0.432	0.401	0.435
	CPM	Pandey	0.341	0.366	0.503	0.398
		simUI	0.342	0.401	0.389	0.415
		simGIC	0.347	0.474	0.391	0.418

HINT-P and BioGrid-AP were partitioned using the four community detection methods: link clustering (Link), BigCLAM, configuration model Modularity Maximization (Config), and Constant Potts model Modularity Maximization (CPM). These communities were divided into four sets using qualitatively similar thresholds as described in the S4 Appendix (Both accepted – "Both acc", only accepted by CommWalker – "CW only", only accepted by functional homogeneity – "FH only", and rejected by both methods – "Both rej"). The presented fractional errors were obtained by computing the overlap between these community set co-expression scores and the co-expression scores of length 6 random walks on the PINs ("overlap score"). Specifically, the fraction of random walk co-expression scores that exceed the 25% quantile threshold of the respective community set co-expression score distribution is calculated. The data shows BioGrid-AP with link clustering and Pandey semantic similarity best captures gene co-expression.

Table S.4: Overlap scores using 10% quantile threshold for data set selection for gene co-expression analysis.

Network	Comm Det	Sem Sim	Both acc	CW only	FH only	Both rej
HINT-P	Link	Pandey	0.368	0.374	0.575	0.411
		simUI	0.365	0.412	0.370	0.424
		simGIC	0.367	0.428	0.438	0.421
	BigClam	Pandey	0.400	0.341	0.510	0.389
		simUI	0.372	0.434	0.384	0.438
		simGIC	0.373	0.585	0.485	0.412
	Config	Pandey	0.455	0.439	0.485	0.405
		simUI	0.415	0.419	0.438	0.416
		simGIC	0.411	0.488	0.392	0.422
	CPM	Pandey	0.441	0.421	0.521	0.400
		simUI	0.403	0.419	0.443	0.423
		simGIC	0.403	0.508	0.430	0.422
BioGrid-AP	Link	Pandey	0.278	0.362	0.486	0.469
		simUI	0.291	0.461	0.431	0.495
		simGIC	0.302	0.444	0.462	0.497
	BigClam	Pandey	0.409	0.422	0.461	0.416
		simUI	0.392	0.451	0.399	0.433
		simGIC	0.394	0.719	0.413	0.456
	Config	Pandey	0.482	0.504	0.598	0.505
		simUI	0.460	0.513	0.506	0.526
		simGIC	0.465	0.555	0.502	0.529
	CPM	Pandey	0.465	0.465	0.511	0.488
		simUI	0.448	0.501	0.463	0.506
		simGIC	0.448	0.571	0.47	0.509

The data was generated as in Table S.3 using the 10% quantile as threshold to calculate the overlap score instead of the 25% quantile. This data confirms that BioGrid-AP with link clustering and Pandey semantic similarity best captures gene co-expression also using the 10% quantil as threshold.

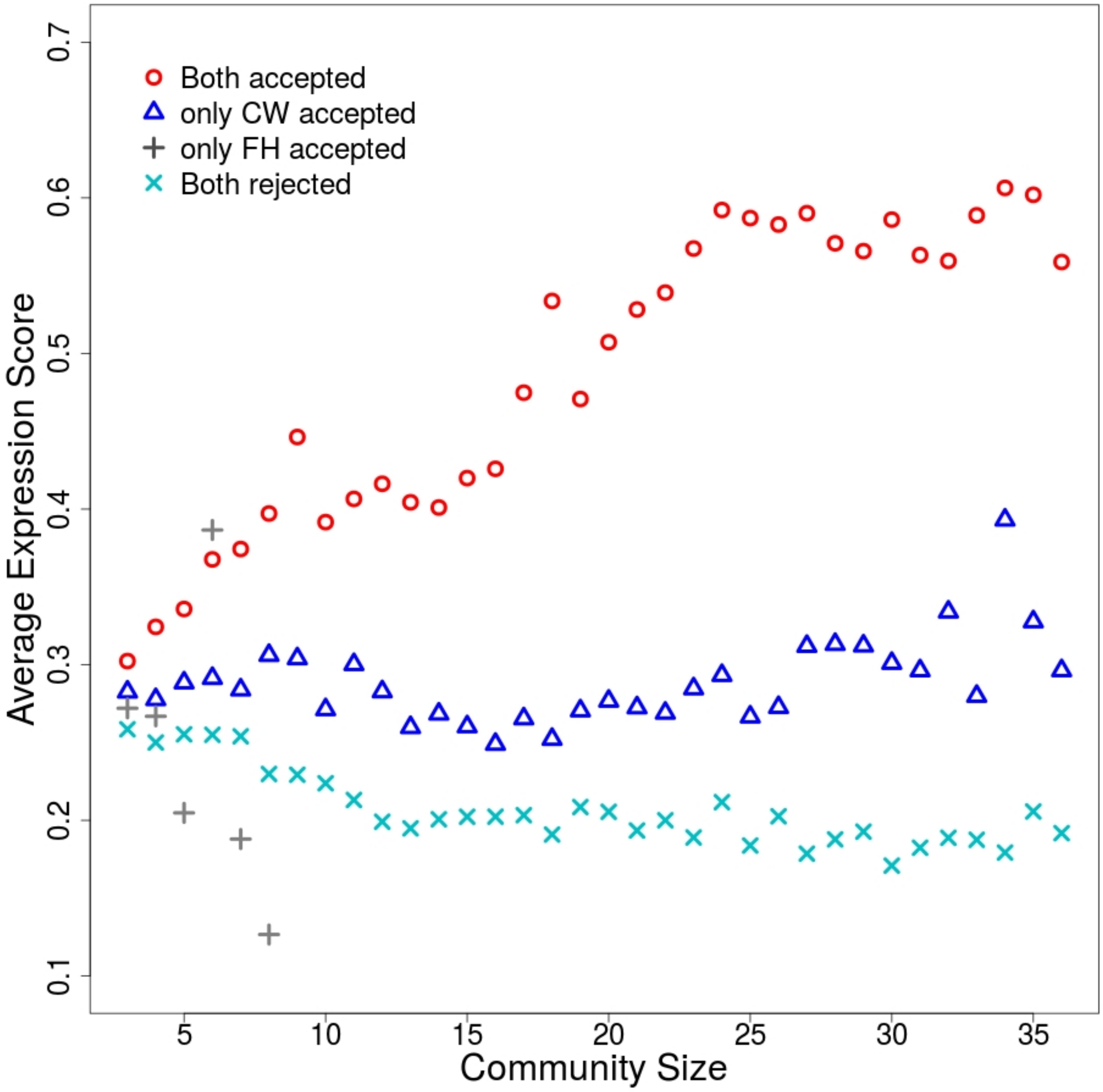

Fig S.29: Comparison of community evaluation methods by average gene co-expression. Link clustering was used to partition BioGrid-AP into communities at multiple resolutions. Using the Pandey measure communities across all resolutions were divided into sets based on whether they were evaluated as functionally significant by both methods ("Both accepted"; red), only CommWalker ("only CW accepted"; blue), only functional homogeneity ("only FH accepted"; grey), or neither ("Both rejected"; turqoise). These sets are compared using their average community expression score (cf. *Methods*) for each community size. The data point marked at community size 36 contains all communities of size > 35. Communities only accepted by CommWalker have higher co-expression scores than communities only accepted by functional homogeneity for most of the size range where both sets are populated. At higher community sizes we can still distinguish the co-expression scores of communities only accepted by CommWalker from those rejected by both methods.

References

Ahn, Y.Y. *et al.* (2010) Link communities reveal multiscale complexity in networks. *Nature*, **466**, 761–4.

Ashburner, M. *et al.* (2000) Gene Ontology: tool for the unification of biology. *Nature Genetics*, **25**, 25–29.

Blondel, V.D. *et al.* (2008) Fast unfolding of communities in large networks. *Journal of Statistical Mechanics: Theory and Experiment*, **2008**, P10008.

Gentleman, R. (2005) Visualizing and Distances Using GO.

Guzzi, P.H. *et al.* (2012) Semantic similarity analysis of protein data: assessment with biological features and issues. *Briefings in Bioinformatics*, **13**, 569–85.

Huang, D.W. *et al.* (2009) Bioinformatics enrichment tools: paths toward the comprehensive functional analysis of large gene lists. *Nucleic Acids Research*, **37**, 1–13.

Lee, H.K. *et al.* (2004) Coexpression analysis of human genes across many microarray data sets. *Genome Research*, **14**, 1085–94.

Lewis, A. *et al.* (2010) The function of communities in protein interaction networks at multiple scales. *BMC Systems Biology*, **4**, 100.

Li, J.J. and Biggin, M.D. (2015) Gene expression. Statistics requantitates the central dogma. *Science*, **347**, 1066–7.

Mazandu, G.K. and Mulder, N.J. (2014) Information Content-Based Gene Ontology Functional Similarity Measures: Which One to Use for a Given Biological Data Type? *PLoS ONE*, **9**, e113859.

Newman, M. and Girvan, M. (2004) Finding and evaluating community structure in networks. *Physical Review E*, **69**, 026113.

Pandey, J. *et al.* (2008) Functional coherence in domain interaction networks. *Bioinformatics*, **24**, i28–34.

Pearson, K. (1907) *On further methods of determining correlation*, volume 4. Dulau and Company.

Pesquita, C. *et al.* (2008) Metrics for GO based protein semantic similarity: a systematic evaluation. *BMC Bioinformatics*, **9 Suppl 5**, S4.

Pesquita, C. *et al.* (2009) Semantic similarity in biomedical ontologies. *PLoS Computational Biology*, **5**, e1000443.

Reichardt, J. and Bornholdt, S. (2006) Statistical mechanics of community detection. *Physical Review E*, **74**, 16110.

Traag, V.A. *et al.* (2011) Narrow scope for resolution-limit-free community detection. *Physical Review E*, **84**, 16114.

van Noort, V. *et al.* (2003) Predicting gene function by conserved co-expression. *Trends in Genetics*, **19**, 238–42.

Yang, J. and Leskovec, J. (2013) Overlapping community detection at scale. In *Proceedings of the Sixth ACM International Conference on Web Search and Data Mining - WSDM '13.* ACM Press, New York, New York, USA, 587.

Zhou, X. *et al.* (2002) Transitive functional annotation by shortest-path analysis of gene expression data. *Proceedings of the National Academy of Sciences of the United States of America*, **99**, 12783–8.
